# Maternal Serum Cytokine Concentrations in Healthy Pregnancy and Preeclampsia

**DOI:** 10.1155/2021/6649608

**Published:** 2021-02-23

**Authors:** Toni Spence, Philip J. Allsopp, Alison J. Yeates, Maria S. Mulhern, J. J. Strain, Emeir M. McSorley

**Affiliations:** Nutrition Innovation Centre for Food and Health (NICHE), School of Biomedical Sciences, Ulster University, Coleraine, Northern Ireland, UK

## Abstract

The maternal immune response is essential for successful pregnancy, promoting immune tolerance to the fetus while maintaining innate and adaptive immunity. Uncontrolled, increased proinflammatory responses are a contributing factor to the pathogenesis of preeclampsia. The Th1/Th2 cytokine shift theory, characterised by bias production of Th2 anti-inflammatory cytokine midgestation, was frequently used to reflect the maternal immune response in pregnancy. This theory is simplistic as it is based on limited information and does not consider the role of other T cell subsets, Th17 and Tregs. A range of maternal peripheral cytokines have been measured in pregnancy cohorts, albeit the changes in individual cytokine concentrations across gestation is not well summarised. Using available data, this review was aimed at summarising changes in individual maternal serum cytokine concentrations throughout healthy pregnancy and evaluating their association with preeclampsia. We report that TNF-*α* increases as pregnancy progresses, IL-8 decreases in the second trimester, and IL-4 concentrations remain consistent throughout gestation. Lower second trimester IL-10 concentrations may be an early predictor for developing preeclampsia. Proinflammatory cytokines (TNF-*α*, IFN-*γ*, IL-2, IL-8, and IL-6) are significantly elevated in preeclampsia. More research is required to determine the usefulness of using cytokines, particularly IL-10, as early biomarkers of pregnancy health.

## 1. Introduction

The importance of the maternal immune system in the establishment and maintenance of successful pregnancy is well researched. The maternal immune system must support immune tolerance to the fetus while maintaining innate and adaptive responses to prevent pathogen invasion. There are key roles for immune cells during pregnancy, particularly uterine natural killer cells (uNK) at the implantation site [1] and at the maternal-fetal interface [2], mediating important processes involved in placentation, for example, angiogenesis. Research exploring the maternal-fetal interface dates back to 1953 when Medawar defined the fetus as an allograft [3] and the uterus was originally proposed to be an immune privileged site [4]. Further theories exist on how the maternal immune response adapts to pregnancy and enables survival of the fetus including the proposed anatomical barrier effect between the mother and fetus [5], maternal systemic and local immune suppression, a lack of major histocompatibility complex (MHC) antigens on fetal tissue, and the maternal Th1/Th2 cytokine shift [6]. Research has focused on the maternal cytokine profile and how it differs during pregnancy, suggesting that a balance between pro- and anti-inflammatory responses is important for optimal pregnancy outcome [7].

Cytokines are signalling proteins directing biological processes throughout pregnancy, from implantation to parturition. The first trimester is a vulnerable stage of pregnancy as complications can be linked back to abnormal placental development [8]. While implantation and placental development are proinflammatory processes, the maternal immune response acts to control inflammation through regulatory and anti-inflammatory mediators [9]. The pregnancy “Th2-like phenomenon” was first described by Wegmann and colleagues, proposing a bias towards Th2 cytokine production to achieve immune tolerance to the fetus [10]. Research using isolated peripheral blood mononuclear cells (PBMCs) from pregnant women between the first and third trimester suggested a “shift” towards Th2 cytokine production during healthy pregnancy compared to nonpregnant women [11]. Following extensive research, the proposed Th1/Th2 gestational cytokine shift was characterised by increased anti-inflammatory Th2 cytokine production in the second trimester [1, 12]. Furthermore, adverse pregnancy complications including preeclampsia have been associated with increased Th1 proinflammatory cytokines [13, 14]. In recent years, however, the Th1/Th2 cytokine profile has been deemed too simplistic. Research now focuses on many contributing mediators of the immune response including regulatory T cells (Tregs) [15] and Th17 cells [16]. A recent review summarised how an imbalance between maternal proinflammatory cytokines and immune regulatory factors (Tregs and IL-10) is a key contributor to preeclampsia [17]. An in-depth understanding of the changes in the maternal cytokine profile could distinguish successful pregnancy from pregnancy complications and help provide a greater understanding of the immune response during pregnancy.

Alterations in the normal immune response during pregnancy may contribute to the onset of pregnancy complications. Hypertensive conditions occur during approximately 10% of pregnancies in the UK with a wide variation in incidence worldwide [18]. In women with normal blood pressure prior to pregnancy, preeclampsia is characterised by hypertension and proteinuria or hypertension with end organ dysfunction with or without proteinuria [19, 20]. The disorder, which develops at ≥20 weeks, is associated with infant complications including fetal growth restriction [21]. Uncontrolled, increased proinflammatory responses alongside less regulatory or anti-inflammatory cytokines are important contributing factors to the pathogenesis of preeclampsia [22], and the maternal cytokine profile has been shown to differ between normotensive pregnancy and preeclampsia [23–25]. Research has shown differences in cytokine concentrations in early pregnancy between healthy women and those who later developed preeclampsia, suggesting that cytokines may be early predictors of preeclampsia [26]. As a result, it is plausible that maternal cytokine concentrations may be potential predictors of adverse pregnancy complications.

Although the maternal immune response is well researched, the change, if any, in individual peripheral cytokines across gestation has not been well summarised. In the past, cytokines have been difficult to detect in maternal plasma [27] and serum [28] from healthy pregnancy, but advances in technology over time has improved the sensitivity of immunoassays used to measure cytokines. Using available data from the current literature, this review was aimed at summarising changes in individual maternal serum cytokine concentrations throughout healthy pregnancy and evaluating associations between maternal serum cytokines and preeclampsia.

## 2. Methods

**Figure 1:**
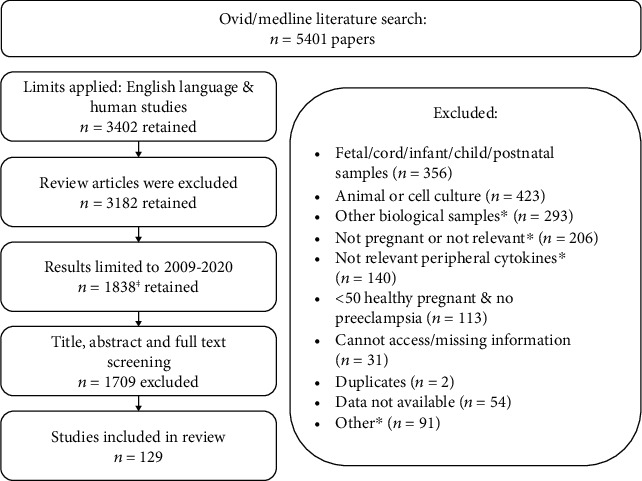
Summary of the literature search and papers retained or excluded. ^ⱡ^Including four additional papers identified from references. ^∗^Other biological samples: e.g., plasma, whole blood, CSF, and PBMCs; not pregnant or not relevant: e.g., IVF, recurrent miscarriage, fertile and infertile women, and infection or periodontitis/gingivitis; not relevant peripheral cytokines: e.g., gene polymorphisms or expression; other included not healthy pregnancy or preeclampsia and adolescent or twin pregnancy.

The research strategy utilised Ovid/Medline databases. Initial literature searches were conducted between 8^th^ May and 7^th^ June 2019. The search was repeated on the 15^th^ April 2020.  Figure 1 summarises the results of our literature search and the papers retained or excluded. Human studies published in the English language on or after 2009 were included if cytokines were measured in maternal serum from a cohort of ≥50 healthy pregnant women or at >1 time point across the three trimesters, regardless of sample size. To obtain data on preeclampsia, the same inclusion criteria were applied with the exception that there was no restriction on sample size.

Exclusion criteria were cytokines measured in other biological samples (e.g., plasma, whole blood, or PBMCs), murine or in vitro models, where there was a different group of participants per sampling time point, and studies which did not report cytokine data (e.g., data were only presented in graphs). Four additional papers were included from the references of studies. Owing to the high volume of papers identified (*n* = 1838), we excluded studies consisting of <50 healthy pregnant women. For this review, cytokine concentrations were obtained from published work, converted to pg/mL for standardisation where possible, expressed as median unless otherwise stated, and reported to 2 decimal places. The gestational age of sample collection is provided where possible based on the mean or median as stated in the original paper and rounded to the nearest whole number. The following cytokines are discussed in this review: IFN-*γ*, TNF-*α*, IL-1*β*, IL-2, IL-4, IL-6, IL-8, IL-10, IL-12, IL-13, IL-17, IL-18, IL-33, and TGF-*β*. The existing data for cytokine concentrations within healthy pregnancy are displayed in Tables 1 and 2 while data for women who developed preeclampsia are in Table 3.

## 3. Cytokines in Healthy Pregnancy

Interferon-gamma (IFN-*γ*) is a proinflammatory cytokine secreted as part of the immune response to damage-associated molecular patterns (DAMPs) or pathogen-associated molecular patterns (PAMPs), by immune cells including natural killer (NK) and Th1 cells [29]. The active protein interacts with a heterodimeric receptor comprising IFN-*γ*R1 and IFN-*γ*R2, resulting in the activation of the JAK-STAT signalling pathway to coordinate immune responses [30]. IFN-*γ* is a key mediator in response to viral pathogens [31, 32] and tumours [33]. At the maternal-fetal interface, IFN-*γ* contributes to the establishment and maintenance of successful pregnancy, mediating endometrial vascular remodelling and angiogenesis [34]. One study observed significant increasing mean concentrations of IFN-*γ* between the first, second, and third trimesters of healthy pregnancy (91.05 pg/mL, 124.50 pg/mL, and 131.05 pg/mL, respectively) [35], while others reported significantly lower IFN-*γ* concentrations in the third trimester compared to the first [36, 37] and second [38] trimesters. Another study detected higher IFN-*γ* concentrations in the third trimester compared to the second [39]. The study participants, however, were overweight and obese which may have influenced their results as obesity is associated with elevated cytokine concentrations including IFN-*γ* [40]. High IFN-*γ* concentrations may also reflect subclinical or asymptomatic infections. Owing to the antiviral activity of IFN-*γ*, controlling for infections is an important factor which restricts comparability of data. While some studies included in this review adjusted for the presence or absence of infection or took infection into consideration when including/excluding participants [38, 41], others did not specify this criterion [39, 42]. Meanwhile, a recent study showed no significant change in maternal serum IFN-*γ* concentrations across 4 time points (10, 12, 19, and 24 weeks) [43]. From the existing data discussed, there is no obvious trend or pattern to the changes in IFN-*γ* and any changes observed may reflect the role of IFN in inflammation owing to infection.

Tumour necrosis factor (TNF)-*α* is a proinflammatory cytokine which is encoded on chromosome 6 and acts through TNF receptors (TNFR1 and TNFR2) expressed by most cells of the immune system [44]. Apart from its key role in inflammatory responses against infection [45], TNF-*α* is an important regulator of normal cell function, influencing vital biological processes including cell proliferation [46], apoptosis [47], and the production of other cytokines such as IL-6 [48]. Apoptosis is a critical process to regulate placenta trophoblast cell survival in normal pregnancy [49]. TNF-*α* binding to TNFR1 can mediate cell death through interactions with the TRAAD adaptor protein and fas-associated death domain (FAAD) or survival through the binding of TRAAD, TNF receptor-associated factor (TRAF), and the receptor interaction protein [50]. In healthy pregnancy, maternal serum TNF-*α* concentrations are significantly higher in the second and third trimesters compared to the first [51]. Another study found that mean TNF-*α* concentrations significantly increased between the first, second, and third trimesters of healthy pregnancy (108.00 pg/mL, 153.01 pg/mL, and 172.89 pg/mL, respectively) [35]. Others have also observed significantly higher TNF-*α* concentrations in the third trimester compared to early pregnancy [52, 53]. Significant increases in maternal serum TNF-*α* concentrations are also reported between the first and second [54, 55] and the second and third trimesters [56]. In contrast, others detected a significant reduction in maternal serum TNF-*α* concentrations between the first and third trimesters [36, 37]. There is also evidence of no change in TNF-*α* between various time points across healthy pregnancy [38, 43, 57–61]. Overall, it is likely that TNF-*α* concentrations increase as gestation progresses albeit not excessively and may support the increased metabolic needs associated with pregnancy.

Interleukin-6 (IL-6) is a pleiotropic cytokine largely produced by monocytes and macrophages but also by other immune and nonimmune cells including T cells and endothelial cells. In the innate immune response, macrophages secrete IL-6 in response to PAMPs which are bound to pattern recognition receptors. When secreted, IL-6 moves to the liver where it stimulates production of acute phase proteins such as C-reactive protein (CRP) [62], thereby promoting inflammation. IL-6 mediates embryo implantation and placental development [63]. Maternal serum IL-6 concentrations significantly increase during healthy pregnancy [64]. Others also reported significantly higher IL-6 concentrations in the third trimester in comparison to samples collected earlier in pregnancy [35, 59, 65, 66]. Repeated sampling in the first half of healthy pregnancy has shown that IL-6 concentrations decrease between the first and second trimesters [43]. In contrast, data from another study suggests the opposite, reporting higher IL-6 concentrations in the second trimester compared to the first trimester [54]. Notably, while several studies in this review found that IL-6 concentrations significantly increase during healthy pregnancy, others reported no significant change [36, 38, 55, 56, 61, 67–70].

Th17 cells are important for coordinating innate and adaptive immune responses against invading pathogens and are involved in the development of autoimmunity. IL-17 (IL-17A) and IL-17F are better understood compared to other members of the IL-17 cytokine family. IL-17 cytokines are secreted by Th17 cells and promote production of other proinflammatory cytokines which, if unregulated, 5can contribute to the development of autoimmune conditions [71]. One study reported that IL-17 concentrations significantly increase with gestation; however, IL-17 was detected in all three trimesters in only three out of 13 women [72], indicating that serum IL-17 may be difficult to detect in healthy pregnancy. A larger study found that IL-17 concentrations significantly decrease between the first and second trimesters of healthy pregnancy [43]. Meanwhile, data from another cohort showed significant variation in IL-17 concentrations across 5 time points throughout healthy pregnancy but reported no “obvious trend” [73]. Owing to few studies measuring maternal serum IL-17 at multiple time points, there is not enough data to understand specific changes during healthy pregnancy. Although not within the scope of this review, recent research examined the importance of the “Th17/Treg” paradigm in pregnancy, whereby the altered Th17 : Treg ratio (reduced Treg cells) may contribute to preeclampsia [16].

Interleukin-1 beta (IL-1*β*) is similar in structure and function to IL-1*α*, and both proteins are encoded by genes located on chromosome 2 and act through binding to the type 1 IL-1 receptor (IL-1R1) to elicit proinflammatory responses [74]. In response to stimuli, including DAMPs or PAMPs, IL-1*β* is secreted by immune cells including monocytes and macrophages [75]. IL-1*β* is a Th1 cytokine but is also associated with Th17 responses [76]. Existing data suggests that IL-1*β* is typically present at low concentrations in maternal serum during healthy pregnancy with fewer studies detecting concentrations above 10 pg/mL [24, 41, 51, 77–79]. A study showed that IL-1*β* is significantly higher in maternal serum within the second and third trimesters compared to the first [51], while others indicate that IL-1*β* significantly decreases between the first and third trimester [36, 37]. In contrast, there is also data supporting no significant change in maternal serum IL-1*β* concentrations across various time points of healthy pregnancy [43, 56]. Such conflicting results may be explained by difficulty in detecting significant changes in IL-1*β* because of its short half-life in circulation [75, 80].

The proinflammatory cytokine, interleukin-2 (IL-2), acts by binding to the IL-2R, influencing T lymphocyte differentiation into effector and memory T cells as well as regulatory T cells which are important for preventing autoimmunity [81]. Evidence suggests that there is a significant reduction in maternal serum IL-2 concentrations between the first and third trimester of healthy pregnancy [36, 37]. The existing data on maternal serum IL-2 concentrations is limited and may be owing to difficulty in detecting the cytokine in healthy pregnancy as studies have noted concentrations below the lower limit of detection (<LLOD) [25, 66, 82]. IL-2 does not appear to be present at high concentrations in healthy pregnancy but is elevated in women who develop complications including preeclampsia [83] which is discussed in more detail later in this review.

The inflammatory chemokine, interleukin-8 (IL-8), signals through binding CXCR1 and CXCR2 to promote recruitment of immune cells such as neutrophils and macrophages to sites of inflammation [84]. IL-8 mediates angiogenesis in vitro [85] which is an important process in pregnancy for fetal development [86]. In healthy pregnancy, IL-8 has been shown to decrease with gestational age during the first half of pregnancy [43] but significantly increase between the second and third trimesters [38]. Another study found higher, albeit nonsignificant, concentrations in the third trimester compared to the second trimester [56]. These changes in IL-8 concentrations may reflect the Th1/Th2 cytokine shift, indicating reduced proinflammatory responses in the second trimester, but the immune profile returns to more proinflammatory responses towards the end of pregnancy. Meanwhile, a study found that IL-8 significantly decreased between the first and third trimesters of healthy pregnancy [36]. While most research has detected significant changes, a smaller study reported no change between the first and third trimesters [37] which may indicate a lack of power within the study design.

IL-12 is a proinflammatory cytokine important in regulating both innate and adaptive immune responses including the differentiation of Th1 cells [87]. Thus, IL-12 plays an important role in the regulation of Th1 immune responses. The IL-12 cytokine consists of two subunits (p35 and p40) encoded by separate genes on chromosomes 3 (IL-12A) and 5 (IL-12B), respectively, and the resulting biologically active heterodimer is IL-12p70 [88]. A significant reduction in maternal serum IL-12 concentrations has been observed between the first and third trimesters of healthy pregnancy [36, 37]. IL-12p70 concentrations are similar between the first and second trimesters [43] and second and third trimesters [66]. Therefore, IL-12 may be lower in the third trimester compared to the first, but because of limited data as no study measured IL-12 within all three trimesters of healthy pregnancy, specific changes are not well understood.

Interleukin-10 (IL-10), encoded on chromosome one, is an anti-inflammatory cytokine and acts through a receptor complex consisting of IL-10R1 and IL-10R2 [89]. The multifunctional cytokine is produced by macrophages, mast cells, Th2 cells, and regulatory T cells (Tregs) and can inhibit proinflammatory cytokines including IFN-*γ* [90]. IL-10 and TGF-*β* are secreted by Tregs, and their immunomodulatory properties control inflammation which is important for successful pregnancy. Tregs are essential for the maintenance of healthy pregnancy, and IL-10 is known to mediate Treg development [91]. In healthy pregnancy, maternal serum IL-10 concentrations significantly decrease between the first and second trimesters [43] and between the second and third trimesters [38], although the difference in concentration values were subtle. Others reported significantly higher IL-10 concentrations in the third trimester compared to the first [51] and second [53]. Notably, there is also evidence of no significant change in IL-10 concentrations during healthy pregnancy [36, 61, 66, 67, 92]. Overall, IL-10 concentrations may increase in the third trimester of healthy pregnancy or remain consistent throughout pregnancy.

IL-4 mediates differentiation of naive T cells into Th2 cells and acts as an anti-inflammatory cytokine by binding to its receptor, IL-4R*α*, and activating the STAT6 signalling pathway [93]. In healthy pregnancy, maternal serum IL-4 concentrations appear to remain constant throughout gestation [43, 53]. Others that have measured IL4 did not look for differences in concentrations across pregnancy [94] while some report that IL-4 is below the LLOD in healthy pregnancy [38, 66]. Although there is limited research, IL-13 [38, 43] and IL-33 [95] concentrations also remain consistent during healthy pregnancy. IL-5, IL-7, IL-9, IL-15, and IL-31 were not discussed in this review due to limited data and/or did not appear to have a relevant role in healthy pregnancy or preeclampsia.

## 4. Serum Cytokines as Early Biomarkers of Preeclampsia

There is increasing interest in the role of cytokines as early biomarkers of preeclampsia. Studies have measured maternal serum cytokines in samples collected prior to the onset of preeclampsia and examined the difference between women who later developed preeclampsia and women who remained healthy. For TNF-*α*, while evidence suggests serum concentrations do not significantly differ between those who later developed preeclampsia and women who remained healthy [42, 58, 77, 96], others report that TNF-*α* measured at 14-18 weeks may be a potential biomarker for the onset of preeclampsia with lower concentrations of TNF-*α* observed in women who later developed preeclampsia [97].

In early pregnancy (10-14 weeks), IL-1*β* significantly differs between women who develop preeclampsia and those who remain healthy (data not in tables as concentration was presented as a multiple of the gestational median value (MoM) ratio) [26]. Furthermore, in maternal samples obtained in the second trimester (approx. 17 weeks), IL-1*β* is reportedly higher in healthy pregnancy compared to women who developed preeclampsia, with higher IL-1*β* concentrations in the second trimester associated with decreased odds of developing preeclampsia [58, 77]. On the other hand, first trimester IL-1*β* concentrations have been linked to preterm birth associated with preeclampsia (<37 weeks) [58], but not with those who only developed preeclampsia [42, 58, 96]. Overall, IL-1*β* may be an early predictor for preeclampsia resulting in preterm delivery albeit more research is needed.

Research has shown no difference in maternal serum IL-8, IL-12, or IL-6 concentrations between women who later developed preeclampsia and women with healthy pregnancy [42, 77]. In the first trimester, a study found significantly higher serum IL-8 concentrations in women who developed preeclampsia but no difference in IL-12p40, IL-12p70, or IL-6 compared to women with healthy pregnancy [96]. In women who went onto developing preeclampsia, another study found that those with samples collected in the first trimester had significantly higher IL-12 and IL-6 concentrations while women with second trimester serum samples had higher IL-8 [58]. From our literature search, there are some data indicating that IL-8, IL-12, and IL-6 may be early predictors for the onset of preeclampsia, but more research would be required to confirm these existing findings.

In samples obtained in the first trimester, some evidence suggests that IL-10 concentrations are significantly higher in women who developed preeclampsia compared to women with healthy pregnancy [65 58], while others indicate no significant difference [42, 96]. In samples collected in the second trimester, IL-10 is significantly lower in women who later develop preeclampsia compared to women who remain healthy [58, 77, 97, 98]. Furthermore, mean IL-10 concentrations at 14-18 weeks are also significantly lower in women who developed severe preeclampsia (21.54 pg/mL) compared to women with mild preeclampsia (14.84 pg/mL) [98]. Another study, however, reported no difference in IL-10 at 14-18 weeks between women who later developed preeclampsia and women who did not [99]. Most studies which reported significant differences had larger cohorts with >50 women who developed preeclampsia [58, 77, 98], while studies that reported no significance consisted of <15 women in the preeclampsia group [49, 96, 99]. It may be important to consider differences in power between these studies. Overall, several studies suggest that lower IL-10 concentrations in the second trimester (14-18 weeks) may be an early predictor for the onset of preeclampsia.

IL-18 concentrations in the second trimester are higher in women who develop preeclampsia compared to controls [77]. Maternal serum TGF-*β* concentrations in the first [58] and second [77] trimesters are higher in women who developed preeclampsia compared to controls. In contrast, another study found that women who developed preeclampsia had lower TGF-*β* concentrations in the second trimester which was significantly associated with preeclampsia [58]. This observation aligns with the studies previously mentioned that reported significantly lower IL-10 concentrations in women who later developed preeclampsia [58, 77, 97, 98], which may reflect the dual importance of Treg cytokines IL-10 and TGF-*β* in successful pregnancy. It is important to consider limitations associated with measuring TGF-*β* in serum as platelets have a large amount of TGF-*β* in their granules [100]; when serum is obtained without anticoagulant platelet, degranulation may occur and result in higher concentrations of TGF-*β*. Overall, lower maternal serum IL-10 concentrations in the second trimester may be associated with the onset of preeclampsia, which may reflect the importance of anti-inflammatory and Treg cytokines in controlling inflammation midgestation.

## 5. Cytokines in Preeclampsia

Cytokines are frequently measured within the third trimester for comparison between women suffering from preeclampsia and healthy pregnant controls. Several studies reported significantly higher IFN-*γ* concentrations in preeclampsia compared to healthy pregnancy [24, 79, 101]. Increased IFN-*γ* concentrations in preeclampsia may reflect increased viral immune responses or natural killer cell activity, as infection is associated with preeclampsia [102] and could subsequently result in the promotion of proinflammatory pathways and pregnancy complications. Others, however, found significantly lower IFN-*γ* concentrations in women with preeclampsia [103] or no significant difference between preeclampsia and healthy pregnant women [104–107]. From the literature discussed in this review, the larger studies found significantly higher IFN-*γ* concentrations [24, 79, 101] and it is likely that IFN-*γ* is elevated during the third trimester in women with preeclampsia compared to healthy pregnant women. The largest study found significantly higher concentrations, but their study design consisted of a wider sampling time period (25-36 weeks) [101].

In the third trimester, several studies report significantly higher maternal serum TNF-*α* concentrations in women with preeclampsia compared to controls [24, 101, 108–115] while others have detected similar or nonsignificantly different concentrations [103, 105, 106, 116]. Variation in study design may have contributed to the differences in findings, for example, sample size, methodology used to measure cytokine concentrations, and exclusion criteria (including maternal infection, preexisting or subclinical illnesses, and smoking). Studies indicating no significant difference in TNF-*α* concentrations between preeclampsia and healthy pregnancy had smaller cohorts (<40 participants) [103, 116] than most studies showing that TNF-*α* is significantly higher in preeclampsia (≥40 participants) [24, 101, 109–112]. The largest study we identified showed higher TNF-*α* concentrations in women with preeclampsia (*n* = 300) than in healthy controls (*n* = 200) excluding smokers and those with urinary or respiratory infections [101], while others who did not find significant differences did not state if they controlled for these factors [103, 105]. Another study observed lower, but not statistically significant, serum TNF-*α* concentrations in women with preeclampsia compared to women with healthy pregnancy at ≥20 weeks [117]. The study consisted of a small sample size (healthy *n* = 24 and preeclampsia *n* = 31) and, as the sampling period crossed between the second and third trimesters, it may have been important to stratify participants by mild/severe- or early/late-onset preeclampsia. Overall, most work indicates that TNF-*α* concentrations are higher in preeclampsia compared to healthy pregnant women, reflecting the enhanced proinflammatory systemic environment.

Maternal serum IL-2 concentrations are significantly higher in preeclampsia compared to healthy pregnant women [24, 79, 101, 114]. Elevated IL-2 concentrations may reflect the proinflammatory environment associated with preeclampsia. Research using a murine model of placental ischemia in pregnancy proposed that IL-2 is a key cytokine mediating natural killer cell activation and placental health in preeclampsia [118]. Others, however, reported no difference in IL-2 concentrations between preeclampsia and healthy pregnancy [103–106].

Multiple studies observed significantly higher IL-8 concentrations in preeclampsia compared to healthy pregnancy [24, 79, 104, 119], while others report no significant difference [25, 103]. Inconsistent findings may be owing to the sampling time point as those who did not find a significant difference collected samples earlier (30-33 weeks) [25, 103] than 37 weeks [24, 79, 119]. Most evidence indicates higher IL-6 concentrations in preeclampsia compared to healthy pregnancy [24, 79, 104, 111, 114, 115, 119–126], while data from other studies show no difference between the two groups [103, 105, 106, 116, 117, 127–129]. From the studies examined in this review, those consisting of larger cohorts have shown significantly higher IL-6 concentrations in preeclampsia compared to controls [24, 79, 111, 115, 125]. Nonetheless, most evidence suggests that IL-8 and IL-6 are higher in preeclampsia compared to healthy pregnant women and may be as a result of preeclampsia.

Research suggests that IL-12p40, IL-12p70 [24, 79], IL-17 [104, 130], IL-18 [24, 79, 131], and TGF-*β* [132, 133] are elevated in women with preeclampsia in the third trimester compared to healthy controls. Others, however, have found no difference in IL-12p70 [25, 104], IL-17 [117, 134], and TGF-*β* [24, 79] in women with preeclampsia compared to controls. In contrast, maternal serum TGF-*β* concentrations are reportedly lower in women with preeclampsia with fetal growth restriction compared to controls [130]. One study reported significantly lower IL-33 concentrations in women with preeclampsia compared to healthy pregnancy in the third trimester [135]. With limited data for these cytokines, more research is required to understand their importance in preeclampsia.

IL-1*β* [24, 79, 103, 110, 136], IL-4 [103, 104, 106, 107], and IL-13 [104, 132] do not appear to significantly contribute to the pathogenesis of preeclampsia as most research examined in this review indicated no difference in these cytokine concentrations between preeclampsia and healthy pregnancy in the third trimester. Others, however, reported that IL-1*β* was significantly higher [104, 123] or lower [25] in women with preeclampsia. Furthermore, other studies suggest that IL-4 is significantly higher in preeclampsia compared to healthy pregnancy [24, 79]. In preeclampsia, the proinflammatory : anti-inflammatory ratio (IL-2: IL-4 and IFN-*γ* : IL-4) is elevated compared to healthy pregnant controls [24], reflecting the more proinflammatory response associated with preeclampsia.

In the third trimester, most studies reported no significant change in maternal serum IL-10 concentrations between women with preeclampsia and healthy pregnancy [25, 97, 103, 104, 106, 117]. In contrast, others have detected significantly higher IL-10 concentrations in preeclampsia compared to healthy pregnant women [24, 79, 107]. As preeclampsia is deemed a more proinflammatory environment, IL-10 concentrations may be raised in response to the inflammatory environment. Another study showed significantly lower IL-10 concentrations in women with preeclampsia compared to controls in the third trimester [137, 138]. As IL-10 is a key cytokine in the regulation of inflammation, lower IL-10 concentrations in women with preeclampsia may suggest that IL-10 is not effectively controlling the proinflammatory environment that occurs during preeclampsia.

Although beyond the scope of this review, the placenta is a key organ contributing to the inflammatory milieu during pregnancy and immune tolerance. Regulation of immune interactions at the maternal-fetal interface is essential, and the placenta microenvironment is biased towards Th2 [139]. Abnormal placenta development is a key factor in the pathogenesis of preeclampsia [140, 141]. It is important to consider that, in preeclampsia, there are increased proinflammatory cytokines in both the maternal circulation and the placenta [17, 138].

## 6. Summary of Findings

**Figure 2:**
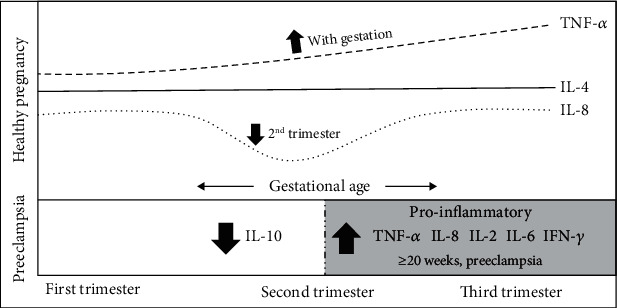
An illustration summarising the changes in the maternal serum cytokine profile in healthy pregnancy and preeclampsia.

We found substantial evidence of changes in cytokine concentrations that occur during healthy pregnancy.  Figure 2 illustrates the main findings of this review. It is likely that TNF-*α* increases as pregnancy progresses, IL-8 decreases in the second trimester, and IL-4 concentrations remain consistent throughout gestation. IFN-*γ* and IL-17 did not appear to have an obvious trend in cytokine concentrations. We found inconsistent or not enough data to specify changes in the remaining cytokines. Lower IL-10 concentrations in early pregnancy may be associated with the later development of preeclampsia, but data collected later in pregnancy were inconsistent. Therefore, it may be important to measure IL-10 in early pregnancy as a predictor of the development of preeclampsia. Most proinflammatory cytokines, particularly TNF-*α*, IFN-*γ*, IL-2, IL-8, and IL-6, are significantly higher in women suffering from preeclampsia compared to women with healthy pregnancy reflecting the enhanced proinflammatory environment.

## 7. Strengths and Limitations

This review was conducted with a systematic approach and covered a large number of research studies in this area. For decades, cytokines have been frequently measured in peripheral maternal samples to examine the immune response. This review provides a summary of the current maternal serum cytokine data, allowing for easy comparison of cytokine concentrations across studies. Limitations of this review were excluding data collected prior to 2009, studies consisting of <50 healthy pregnant women, and cytokines measured in other biological samples. Comparability of data between studies may be limited by differences in study design including varying methods used to detect cytokine concentrations with different specificity and sensitivity limits (ELISA vs. multiplex immunoassays), sample collection, sample storage conditions, and duration.

## 8. Future Directions

Although maternal serum cytokine concentrations have been measured in numerous cohorts, we found limited data for several cytokines. Many studies obtained samples at one or two trimesters of pregnancy which limited our ability to determine change across healthy gestation for many cytokines. As cytokines are a network of signalling proteins which influence each other as well as their target cells, obtaining a range of biological samples at multiple time points across healthy gestation will allow for in-depth analysis on changes in maternal cytokine concentrations. In future, more sensitive methods, e.g., flow cytometry analysis, may be used alongside peripheral cytokine assays to provide a better indication of cell subtypes and intracellular cytokine expression. It may also be useful to examine further if IL-10 measured in early pregnancy is a good predictor for the development of preeclampsia.

## Figures and Tables

**Table 1:** Inflammatory cytokines measured in maternal serum in healthy pregnancy.

	N.	Concentration units			Trimester 1		Trimester 2		Trimester 3	Ref.
				GA	Concentration	GA	Concentration	GA	Concentration	
IFN-*γ*	82	mean (SD)	pg/mL	UR	91.05 (8.50)	UR	124.50 (9.50)	UR	131.05 (11.30)	[35]
IFN-*γ*	168	mean (SE)	pg/mL	7	8.20 (1.30)	20	8.80 (1.20)			[94]
IFN-*γ*	71	median (range)	pg/mL	7-10	87.90 (15.40-567.60)					[142]
IFN-*γ*	77	median(25^th^, 75^th^ percentile)	pg/mL	10	9.10 (3.90, 19.00)					[96]
IFN-*γ*	250	median (IQR)	pg/mL	11	4.00 (6.00)					[143]
IFN-*γ*	178	median (range)	pg/mL	11	4.00 (4.00-4000.00)					[58]
IFN-*γ*	504	median(25^th^, 75^th^ percentile)	pg/mL	11-13	434.00 (371.00, 501.00)					[42]
IFN-*γ*	104	median(25^th^, 75^th^ percentile)	pg/mL	10	54.60 (43.30, 66.40)					[43]
IFN-*γ*	96	median(25^th^, 75^th^ percentile)	pg/mL	12	53.40 (45.30, 66.40)					[43]
IFN-*γ*	93	median(25^th^, 75^th^ percentile)	pg/mL			19	52.20 (45.50, 61.00)			[43]
IFN-*γ*	101	median(25^th^, 75^th^ percentile)	pg/mL			24	52.60 (41.30, 60.70)			[43]
IFN-*γ*	392	median (IQR)	pg/mL			17	4.00 (4.00)			[143]
IFN-*γ*	233	median (range)	pg/mL			17	4.00 (4.00-2117.00)			[58]
IFN-*γ*	162	mean (SE)	pg/mL			14-18	1.67 (0.08)			[99]
IFN-*γ*	105	mean (SD)	pg/mL			18	12.48 (4.13)			[144]
IFN-*γ*	<139	median	pg/mL			15-19	259.00			[145]
IFN-*γ*	204	mean (SD)	log-pg/mL			14-18	0.73 (2.34)			[99]
IFN-*γ*	297	median (range)	pg/mL			16	57.00 (4.00-4000.00)			[77]
IFN-*γ*	>700	median (range)	pg/mL			15-20	4.85 (0.05-324.21)			[75]
IFN-*γ*	78	median (IQR)	pg/mL			12-16	3.03 (1.40-7.59)			[146]
IFN-*γ*	200	mean (SEM)	pg/mL			25-36	28.48 (0.97)			[101]
IFN-*γ*	87	mean (SD)	pg/mL			23	0.79 (0.33)	33	0.70 (0.33)	[38]
IFN-*γ*	44	median (IQR)	pg/mL			15	128.90 (79.00-220.80)	32	184.90 (101.30-241.70)	[39]
IFN-*γ*	44	median (IQR)	pg/mL			24	168.20 (129.40-239.60)			[39]
IFN-*γ*	56	median(25^th^, 75^th^ percentile)	pg/mL	9-11	3.40 (0.00, 29.00)			29-31	2.00 (0.10, 55.50)	[36]
IFN-*γ*	28	median (25^th^, 75^th^ percentile)	pg/mL	9-11	3.40 (1.70, 6.10)			29-31	1.50 (1.00,3.30)	[37]
IFN-*γ*	57	mean (SD)	pg/mL			24	5.24 (21.40)			[147]
IFN-*γ*	55	mean (SD)	pg/mL					36	3.13 (5.77)	[147]
IFN-*γ*	64	median (IQR)	pg/mL					31	6.30 (9.70)	[148]
IFN-*γ*	60	median (25^th^, 75^th^ percentile)	pg/mL					36	3.00 (2.00, 3.00)	[24]
IFN-*γ*	50	median	pg/mL					38	217.10	[105]
IFN-*γ*	50	mean (SEM)	pg/mL					37	221.00 (15.40)	[106]
IFN-*γ*	60	median (IQR)	pg/mL					36	3.00 (2.00-3.00)	[79]
IFN-*γ*	1158	mean (SD)	pg/mL					28	5.63 (20.26)	[149]
IFN-*γ*	718	mean (SD)	pg/mL					28	5.43 (20.30)	[150]
IFN-*γ*	292	median (IQR)	pg/mL					39	27.89 (16.04-48.29)	[151]

TNF-*α*	≤22	median (range)	pg/mL	≤14	2.11 (1.45-4.10)	15-27	2.09 (0.69-34.70)	≥28	2.28 (1.28-5.40)	[57]
TNF-*α*	46	median (IQR)	pg/mL	15	60.10 (46.70-97.90)	24	91.30 (66.30-134.70)	32	90.50 (45.40-122.30)	[51]
TNF-*α*	104	median	pg/mL	12	11.20	19	16.10	29	15.30	[152]
TNF-*α*	77	median (IQR)	pg/mL	12	2.10 (1.90-2.40)	20	2.30 (2.00-2.60)	29	2.30 (2.00-2.50)	[55]
TNF-*α*	82	mean (SD)	pg/mL	UR	108.00 (11.38)	UR	153.01 (11.82)	UR	172.89 (19.48)	[35]
TNF-*α*	20	mean (SD)	pg/mL	UR	11.37 (9.01)	UR	21.44 (35.29)	UR	24.48 (42.33)	[53]
TNF-*α*	168	mean (SE)	pg/mL	7	459.20 (85.00)	20	415.40 (49.10)			[94]
TNF-*α*	94	mean (SD)	pg/mL	12	5.15 (3.26)					[147]
TNF-*α*	77	median (25^th^, 75^th^ percentile)	pg/mL	10	10.90 (7.70, 13.90)					[96]
TNF-*α*	800	median (IQR)	pg/mL	11-13	1.371 (1.288-1.46)					[153]
TNF-*α*	504	median (25^th^, 75^th^ percentile)	pg/mL	11-13	65.50 (57.10, 76.40)					[42]
TNF-*α*	71	median (range)	pg/mL	7-10	0.00 (0.00-97.31)					[142]
TNF-*α*	27	median (IQR)	pg/mL	7-10	0.95 (0.88-1.14)	16-20	1.08 (0.95-1.33)			[54]
TNF-*α*	178	median (range)	pg/mL	11	4.00 (4.00-754.00)					[58]
TNF-*α*	104	median (25^th^, 75^th^ percentile)	pg/mL	10	24.10 (20.40, 29.90)					[43]
TNF-*α*	96	median (25^th^, 75^th^ percentile)	pg/mL	12	23.50 (20.40, 28.70)					[43]
TNF-*α*	93	median (25^th^, 75^th^ percentile)	pg/mL			19	24.20 (20.30, 29.20)			[43]
TNF-*α*	101	median (25^th^, 75^th^ percentile)	pg/mL			24	22.80 (20.60, 29.30)			[43]
TNF-*α*	78	median (IQR)	pg/mL			12-16	6.20 (3.74-12.15)			[145]
TNF-*α*	100	mean (SD)	pg/mL			16	1.30 (1.00)			[154]
TNF-*α*	297	median (range)	pg/mL			16	4.00 (4.00-4000.00)			[77]
TNF-*α*	233	median (range)	pg/mL			17	4.00 (4.00-930.00)			[58]
TNF-*α*	<139	median	pg/mL			15-19	62.20			[145]
TNF-*α*	204	mean (SD)	log pg/mL			14-18	2.18 (1.52)			[99]
TNF-*α*	162	mean (SE)	pg/mL			14-18	601.37 (63.54)			[97]
TNF-*α*	621	median (IQR)	pg/mL			14	4.60 (2.91-7.57)	28	4.65 (3.09-7.71)	[68]
TNF-*α*	156	mean (SD)	pg/mL			14-18	1.30 (1.05)	28-32	1.34 (0.84)	[155]
TNF-*α*	171	mean (SD)	pg/mL			18	0.93 (1.50)	32	1.20 (1.60)	[156]
TNF-*α*	171	mean (SD)	pg/mL			18	1.16 (2.33)	32	1.24 (1.91)	[69]
TNF-*α*	171	mean (SD)	pg/mL			18	0.99 (1.38)	32	1.21 (1.30)	[69]
TNF-*α*	145	Mean (SD)	pg/mL			18	0.44 (0.41)	32	0.55 (0.45)	[59]
TNF-*α*	>700	median (range)	pg/mL			15-20	4.68 (0.22-347.24)			[78]
TNF-*α*	57	mean (SD)	pg/mL			22-23	3.44 (0.23)			[157]
TNF-*α*	138	mean (SD)	pg/mL			23	10.00 (3.20)			[158]
TNF-*α*	227	mean (SEM)	pg/mL			24-28	10.90 (0.30)			[159]
TNF-*α*	65	median (IQR)	pg/mL			24-28	42.37 (70.12)			[160]
TNF-*α*	178	mean (SD)	pg/mL			21	6.69 (7.61)			[161]
TNF-*α*	167	mean (SD)	pg/mL			21	7.20 (7.20)			[162]
TNF-*α*	85	median (IQR)	pg/mL			21	1.15 (0.79-1.79)			[163]
TNF-*α*	60	mean (SD)	pg/mL			22	7.21 (1.19)			[164]
TNF-*α*	93	median (IQR)	pg/mL			23	0.09 (0.09,0.55)			[165]
TNF-*α*	137	mean (SD)	pg/mL			24	4.89 (2.44)			[147]
TNF-*α*	98	median (IQR)	pg/mL			24-28	0.20 (0.130-0.310)			[166]
TNF-*α*	84	mean (SD)	pg/mL			26	2.10 (0.30)			[167]
TNF-*α*	100	mean (SD)	pg/mL			27	2.05 (0.28)			[168]
TNF-*α*	200	mean (SEM)	pg/mL			25-36	97.60 (10.15)			[101]
TNF-*α*	20	median (range)	pg/mL			23-25	1.10 (0.10-1.50)	32-36	1.20 (0.10-2.10)	[66]
TNF-*α*	202	mean (SD)	pg/mL			13	8.22 (3.59)	30	9.22 (4.39)	[52]
TNF-*α*	202	mean (SD)	pg/mL			21	8.51 (3.75)			[52]
TNF-*α*	87	mean (SD)	log pg/mL			23	0.35 (0.12)	33	0.35 (0.12)	[38]
TNF-*α*	71	mean (SD)	pg/mL			28	7.30 (1.80)	37	7.80 (1.90)	[56]
TNF-*α*	32	median (IQR)	pg/mL	8-12	9.87 (8.59-12.00)			28-32	10.00 (8.56-13.15)	[61]
TNF-*α*	56	median (25^th^, 75^th^ percentile)	pg/mL	9-11	11.50 (1.40, 104.60)			29-31	10.20 (0.70, 72.90)	[36]
TNF-*α*	28	median (25^th^, 75^th^ percentile)	pg/mL	9-11	11.00 (5.80, 26.90)			29-31	7.60 (4.50, 15.00)	[37]
TNF-*α*	439	median (IQR)	pg/mL					30	5.67 (3.16)	[169]
TNF-*α*	231	median (IQR)	pg/mL					32	0.29 (0.09,0.78)	[165]
TNF-*α*	1,494	median (25^th^, 75^th^ percentile)	pg/mL					32	3.50 (2.30, 5.00)	[170]
TNF-*α*	774	median (IQR)	pg/mL					33	3.40 (2.50)	[171]
TNF-*α*	564	median (25^th^, 75^th^ percentile)	pg/mL					30	5.50 (3.90, 10.50)	[172]
TNF-*α*	62	median (range)	pg/mL					34	3.97 (3.06-16.30)	[115]
TNF-*α*	<55	mean (SD)	pg/mL					28-36	16.04 (0.69)	[138]
TNF-*α*	<139	mean (SD)	pg/mL					>37	15.30 (0.61)	[138]
TNF-*α*	195	geometric mean (95% CI)	pg/mL					39	1.96 (1.71-2.24)	[173]
TNF-*α*	64	median (IQR)	pg/mL					31	4.50 (7.50)	[148]
TNF-*α*	50	mean (SD)	pg/mL					31	2.27 (0.85)	[174]
TNF-*α*	50	median	pg/mL					38	198.80	[105]
TNF-*α*	50	mean (SEM)	pg/mL					37	186.00 (15.60)	[106]
TNF-*α*	78	median (IQR)	pg/mL					28-41	2.67 (1.51-5.51)	[111]
TNF-*α*	60	median (IQR)	pg/mL					36	2.00 (1.00-2.00)	[79]
TNF-*α*	60	mean (SD)	pg/mL					30	10.10 (3.20)	[175]
TNF-*α*	60	median (25^th^, 75^th^ percentile)	pg/mL					36	2.00 (1.00, 2.00)	[24]
TNF-*α*	133	mean (SD)	pg/mL					36	5.56 (4.35)	[147]
TNF-*α*	1158	mean (SD)	pg/mL					28	7.26 (7.55)	[149]
TNF-*α*	718	mean (SD)	pg/mL					28	5.98 (8.54)	[150]
TNF-*α*	63	mean (SD)	pg/mL					33	0.81 (0.48)	[176]
TNF-*α*	50	mean (SEM)	pg/mL					30-40	46.00 (2.40)	[114]
TNF-*α*	52	mean (SD)	pg/mL					UR	4.12 (2.32)	[177]

IL-1*β*	46	median (IQR)	pg/mL	15	49.20 (0.10-85.00)	24	69.50 (0.10-104.40)	32	62.00 (0.10-109.00)	[51]
IL-1*β*	71	median (range)	pg/mL	7-10	0.85 (0.00-55.44)					[142]
IL-1*β*	77	median (25^th^, 75^th^ percentile)	pg/mL	10	1.70 (1.00, 3.10)					[96]
IL-1*β*	504	median (25^th^, 75^th^ percentile)	pg/mL	11-13	2.59 (2.24, 3.04)					[42]
IL-1*β*	250	median (IQR)	pg/mL	11	9.00 (12.00)					[143]
IL-1*β*	178	median (range)	pg/mL	11	9.00 (4.00-1489.00)					[58]
IL-1*β*	83	mean (SD)	pg/mL	12	1.13 (4.21)					[147]
IL-1*β*	104	median (25^th^, 75^th^ percentile)	pg/mL	10	1.60 (1.20, 2.00)					[43]
IL-1*β*	96	median (25^th^, 75^th^ percentile)	pg/mL	12	1.60 (1.10, 1.90)					[43]
IL-1*β*	93	median (25^th^, 75^th^ percentile)	pg/mL			19	1.50 (1.20, 1.90)			[43]
IL-1*β*	101	median (25^th^, 75^th^ percentile)	pg/mL			24	1.50 (1.20, 1.90)			[43]
IL-1*β*	78	median (IQR)	pg/mL			12-16	1.55 (1.00-6.61)			[146]
IL-1*β*	100	mean (SD)	pg/mL			16	1.90 (1.00)			[154]
IL-1*β*	297	median (range)	pg/mL			16	272.00 (4.00-4000.00)			[77]
IL-1*β*	392	median (IQR)	pg/mL			17	10.00 (13.00)			[143]
IL-1*β*	233	median (range)	pg/mL			17	10.00 (4.00-373.00)			[58]
IL-1*β*	>700	median (range)	pg/mL			15-20	12.45 (0.03-13258.28)			[78]
IL-1*β*	178	mean (SD)	pg/mL			21	0.57 (0.31)			[161]
IL-1*β*	167	mean (SD)	pg/mL			21	0.80 (0.50)			[162]
IL-1*β*	60	mean (SD)	pg/mL			22	0.92 (0.51)			[164]
IL-1*β*	57	mean (SD)	pg/mL			22-23	9.25 (0.71)			[157]
IL-1*β*	138	mean (SD)	pg/mL			23	0.70 (0.60)			[158]
IL-1*β*	108	mean (SD)	pg/mL			24	0.92 (1.75)			[147]
IL-1*β*	71	mean (SD)	pg/mL			28	0.80 (0.60)	37	0.90 (0.70)	[56]
IL-1*β*	56	median (25^th^, 75^th^ percentile)	pg/mL	9-11	1.00 (0.00, 10.30)			29-31	0.60 (0.00, 70.80)	[36]
IL-1*β*	28	median (25^th^, 75^th^ percentile)	pg/mL	9-11	0.80 (0.50, 1.40)			29-31	0.50 (0.30, 0.90)	[37]
IL-1*β*	1158	mean (SD)	pg/mL					28	0.32 (0.62)	[149]
IL-1*β*	718	mean (SD)	pg/mL					28	0.26 (0.58)	[150]
IL-1*β*	439	median (IQR)	pg/mL					30	0.48 (0.42)	[169]
IL-1*β*	564	median (10^th^, 90^th^ percentile)	pg/mL					30	0.50 (0.20, 1.30)	[172]
IL-1*β*	64	median (IQR)	pg/mL					31	0.40 (0.80)	[148]
IL-1*β*	1,494	median (25^th^, 75^th^ percentile)	pg/mL					32	1.20 (0.20, 6.80)	[170]
IL-1*β*	774	median (IQR)	pg/mL					33	1.10 (5.00)	[171]
IL-1*β*	63	mean (SD)	pg/mL					33	0.51 (0.24)	[176]
IL-1*β*	≤117	median (range)	pg/mL					30-33	0.60 (0.02-3.54)	[25]
IL-1*β*	195	geometric mean (95% CI)	pg/mL					39	2.12 (1.91-2.35)	[173]
IL-1*β*	60	median (IQR)	pg/mL					36	27.00 (23.00-31.50)	[79]
IL-1*β*	60	median (25^th^, 75^th^ percentile)	pg/mL					36	27.00 (23.00, 31.50)	[24]
IL-1*β*	94	mean (SD)	pg/mL					36	0.80 (1.94)	[147]
IL-1*β*	52	mean (SD)	pg/mL					UR	3.50 (5.46)	[177]

IL-2	71	median (range)	pg/mL	7-10	2.58 (0.00-301.90)					[142]
IL-2	77	median (25^th^, 75^th^ percentile)	pg/mL	10	3.10 (3.10, 3.20)					[96]
IL-2	504	median (25^th^, 75^th^ percentile)	pg/mL	11-13	18.30 (16.10, 21.20)					[42]
IL-2	104	median (25^th^, 75^th^ percentile)	pg/mL	10	1.70 (0.60, 3.40)					[43]
IL-2	96	median (25^th^, 75^th^ percentile)	pg/mL	12	1.60 (0.70, 2.90)					[43]
IL-2	93	median (25^th^, 75^th^ percentile)	pg/mL			19	1.10 (0.40, 3.00)			[43]
IL-2	101	median (25^th^, 75^th^ percentile)	pg/mL			24	0.80 (0.20, 2.00)			[43]
IL-2	>700	median (range)	pg/mL			15-20	0.38 (0.01-29.37)			[78]
IL-2	78	median (IQR)	pg/mL			12-16	6.01 (1.34-27.31)			[146]
IL-2	200	mean (SEM)	pg/mL			25-36	71.90 (0.76)			[101]
IL-2	56	median (25^th^, 75^th^ percentile)	pg/mL	9-11	3.50 (0.00, 39.80)			29-31	3.00 (0.00, 206.00)	[36]
IL-2	28	median (25^th^, 75^th^ percentile)	pg/mL	9-11	4.00 (1.60, 7.30)			29-31	3.40 (0.50, 6.40)	[37]
IL-2	1158	mean (SD)	pg/mL					28	0.29 (0.60)	[149]
IL-2	718	mean (SD)	pg/mL					28	0.24 (0.60)	[150]
IL-2	64	median (IQR)	pg/mL					31	1.20 (6.30)	[148]
IL-2	50	median	pg/mL					38	274.40	[105]
IL-2	60	median (IQR)	pg/mL					36	4.00 (4.00-5.00)	[79]
IL-2	60	median (25^th^, 75^th^ percentile)	pg/mL					36	4.00 (4.00, 5.00)	[24]
IL-2	50	mean (SEM)	pg/mL					37	274.00 (1.24)	[106]
IL-2	50	mean (SEM)	pg/mL					30-40	79.00 (1.04)	[114]

IL-6	46	median (IQR)	pg/mL	15	0.10 (0.10-2.70)	24	0.10 (0.10-6.60)	32	0.10 (0.10-7.80)	[51]
IL-6	104	median	pg/mL	12	0.60	19	3.00	29	1.90	[152]
IL-6	77	median (IQR)	pg/mL	12	0.71 (0.49-1.13)	20	0.75 (0.47-1.20)	29	0.77 (0.53-1.19)	[55]
IL-6	75	mean (SD)	pg/mL	9-17	0.77 (0.38)	18-24	0.93 (0.58)	29-35	1.11 (0.61)	[64]
IL-6	82	mean (SD)	pg/mL	UR	53.08 (8.90)	UR	58.00 (19.08)	UR	78.33 (17.08)	[35]
IL-6	103	median (IQR)	pg/mL	11-14	1.00 (1.00)	24-28	2.00 (2.00)	30-34	2.00 (3.00)	[65]
IL-6	158	mean (SD)	pg/mL	5-12	1.78 (5.06)					[178]
IL-6	71	median (range)	pg/mL	7-10	6.59 (1.23-53.56)					[142]
IL-6	77	median (25^th^, 75^th^ percentile)	pg/mL	10	3.10 (2.30, 5.20)					[96]
IL-6	250	median (IQR)	pg/mL	11	73.50 (197.00)					[143]
IL-6	178	median (range)	pg/mL	11	63.00 (4.00-4000.00)					[58]
IL-6	94	median (SD)	pg/mL	11-14	1.50 (1.01)					[179]
IL-6	504	median (25^th^, 75^th^ percentile)	pg/mL	11-13	9.28 (7.98, 10.88)					[42]
IL-6	80	mean (SD)	pg/mL	12	17.00 (72.50)					[147]
IL-6	27	median (IQR)	pg/mL	7-10	1.17 (0.75-1.69)	16-20	1.55 (0.96-3.01)			[54]
IL-6	104	median (25^th^, 75^th^ percentile)	pg/mL	10	1.40 (0.80, 2.10)					[43]
IL-6	96	median (25^th^, 75^th^ percentile)	pg/mL	12	1.30 (0.80, 1.80)					[43]
IL-6	93	median (25^th^, 75^th^ percentile)	pg/mL			19	1.10 (0.60, 1.70)			[43]
IL-6	101	median (25^th^, 75^th^ percentile)	pg/mL			24	1.10 (0.70, 1.80)			[43]
IL-6	392	median (IQR)	pg/mL			17	4.00 (209.00)			[143]
IL-6	233	median (range)	pg/mL			17	71.00 (4.00-4000.00)			[58]
IL-6	22	mean (SD)	pg/mL	8-12	1.40 (1.30)			28-34	2.20 (3.20)	[67]
IL-6	32	median (IQR)	pg/mL	8-12	0.00 (0.00-2.09)			28-32	0.00 (0.00-2.23)	[61]
IL-6	56	median (25^th^, 75^th^ percentile)	pg/mL	9-11	5.50 (1.80, 10.40)			29-31	4.80 (2.20, 9.20)	[36]
IL-6	28	Median (25^th^, 75^th^ percentile)	pg/mL	9-11	6.00 (2.20, 11.80)			29-31	4.80 (2.90, 9.50)	[37]
IL-6	621	median (IQR)	pg/mL			14	9.48 (4.38-26.26)	28	9.37 (4.21-22.34)	[68]
IL-6	78	median (IQR)	pg/mL			12-15	1.92 (1.57-2.56)			[180]
IL-6	100	mean (SD)	pg/mL			16	4.30 (3.20)			[154]
IL-6	297	median (range)	pg/mL			16	4.00 (4.00-4000.00)			[77]
IL-6	<139	median	pg/mL			15-19	13.20			[145]
IL-6	>700	median (range)	pg/mL			15-20	13.16 (0.09-8045.24)			[78]
IL-6	91	mean (SD)	pg/mL			21	4.00 (2.10)			[181]
IL-6	178	mean (SD)	pg/mL			21	3.76 (2.08)			[161]
IL-6	167	mean (SD)	pg/mL			21	3.90 (2.10)			[162]
IL-6	60	mean (SD)	pg/mL			22	3.91 (1.66)			[164]
IL-6	57	mean (SD)	pg/mL			22-23	2.57 (0.20)			[157]
IL-6	138	mean (SD)	pg/mL			23	1.90 (1.40)			[158]
IL-6	130	mean (SD)	pg/mL			24	13.00 (32.80)			[147]
IL-6	227	mean (SEM)	pg/mL			24-28	2.60 (0.20)			[159]
IL-6	65	mean (SD)	pg/mL			UR	1.70 (0.84)			[182]
IL-6	98	median (IQR)	pg/mL			24-28	0.16 (0.100-0.33)			[166]
IL-6	21	mean (SD)	pg/mL			16-27	1.64 (0.02-14.42)	28-40	0.01 (0.00-0.02)	[120]
IL-6	171	mean (SD)	pg/mL			18	2.17 (1.70)	32	2.60 (1.80)	[156]
IL-6	71	mean (SD)	pg/mL			28	1.80 (1.40)	37	2.00 (1.00)	[56]
IL-6	20	median (range)	pg/mL			23-25	2.00 (1.10-3.40)	32-36	2.70 (1.50-9.80)	[66]
IL-6	171	mean (SD)	pg/mL			18	2.49 (1.85)	32	2.69 (2.02)	[69]
IL-6	171	mean (SD)	pg/mL			18	1.81 (1.33)	32	2.37 (1.46)	[69]
IL-6	145	mean (SD)	pg/mL			18	0.99 (0.38)	32	1.16 (0.37)	[59]
IL-6	45	median (IQR)	pg/mL			24-27	0.89 (0.75-1.07)	29-32	1.02 (0.83-1.22)	[70]
IL-6	87	mean (SD)	log pg/mL			23	0.20 (0.14)	33	0.22 (0.14)	[38]
IL-6	156	mean (SD)	pg/mL			14-18	8.145 (1.55)	28-32	8.28 (1.57)	[155]
IL-6	1158	mean (SD)	pg/mL					28	0.96 (1.44)	[149]
IL-6	718	mean (SD)	pg/mL					28	0.83 (1.40)	[150]
IL-6	<55	mean (SD)	pg/mL					28-36	17.07 (0.44)	[138]
IL-6	<139	mean (SD)	pg/mL					>37	16.12 (0.33)	[138]
IL-6	439	median (IQR)	pg/mL					30	1.11 (0.67)	[169]
IL-6	1,494	median (25^th^, 75^th^ percentile)	pg/mL					32	1.30 (0.50, 3.80)	[170]
IL-6	774	median (IQR)	pg/mL					33	1.10 (2.90)	[171]
IL-6	63	mean (SD)	pg/mL					33	1.33 (1.76)	[176]
IL-6	564	median (10^th^, 90^th^ percentile)	pg/mL					30	1.10 (0.70, 2.30)	[172]
IL-6	64	median (IQR)	pg/mL					31	3.10 (13.10)	[148]
IL-6	50	mean (SD)	log pg/mL					31	0.33 (0.46)	[174]
IL-6	82	median (IQR)	pg/mL					24-31	0.80 (0.50-1.10)	[183]
IL-6	50	median	pg/mL					38	149.80	[105]
IL-6	78	median (IQR)	pg/mL					28-41	1.82 (1.52-2.04)	[111]
IL-6	62	median (range)	pg/mL					34	13.90 (2.20-35.40)	[115]
IL-6	97	mean (SD)	pg/mL					35	1.68 (1.04)	[184]
IL-6	60	median (IQR)	pg/mL					36	7.00 (5.00-9.00)	[79]
IL-6	60	median (25^th^, 75^th^ percentile)	pg/mL					36	7.00 (5.00, 9.00)	[24]
IL-6	74	median (range)	pg/mL					36	10.90 (0.61-65.70)	[125]
IL-6	68	median (range)	pg/mL					36	11.11 (0.02-65.77)	[121]
IL-6	104	mean (SD)	pg/mL					36	18.90 (65.80)	[147]
IL-6	50	mean (SEM)	pg/mL					37	154.00 (28.10)	[106]
IL-6	50	mean (SD)	pg/mL					37	9.00 (6.49)	[185]
IL-6	289	median (IQR)	pg/mL					39	4.06 (2.08-10.48)	[151]
IL-6	52	mean (SD)	pg/mL					UR	6.37 (17.43)	[177]
IL-6	50	mean (SEM)	pg/mL					30-40	2.80 (0.24)	[114]

IL-8	71	median (range)	pg/mL	7-10	4.40 (0.00-62.60)					[142]
IL-8	77	median (25^th^, 75^th^ percentile)	pg/mL	10	87.80 (40.34, 195.00)					[96]
IL-8	504	median (25^th^, 75^th^ percentile)	pg/mL	11-13	18.00 (15.60, 21.50)					[42]
IL-8	250	median (IQR)	pg/mL	11	19.00 (101.00)					[143]
IL-8	178	median (range)	pg/mL	11	21.50 (4.00-4000.00)					[58]
IL-8	87	mean (SD)	pg/mL	12	248.20 (372.20)					[147]
IL-8	104	median (25^th^, 75^th^ percentile)	pg/mL	10	5.50 (4.50, 7.00)					[43]
IL-8	96	median (25^th^, 75^th^ percentile)	pg/mL	12	5.40 (4.30, 6.60)					[43]
IL-8	93	median (25^th^, 75^th^ percentile)	pg/mL			19	5.30 (4.10, 6.30)			[43]
IL-8	101	median (25^th^, 75^th^ percentile)	pg/mL			24	5.20 (4.10, 6.10)			[43]
IL-8	78	median (IQR)	pg/mL			12-16	561.90 (65.31-3071.00)			[146]
IL-8	297	median (range)	pg/mL			16	4.00 (4.00-4000.00)			[77]
IL-8	100	mean (SD)	pg/mL			16	11.00 (9.40)			[154]
IL-8	392	median (IQR)	pg/mL			17	18.00 (113.00)			[143]
IL-8	233	median (range)	pg/mL			17	15.00 (4.00-4000.00)			[58]
IL-8	>700	median (range)	pg/mL			15-20	1223.89 (7.69-20182.15)			[78]
IL-8	60	mean (SD)	pg/mL			22	0.74 (0.12)			[164]
IL-8	138	mean (SD)	pg/mL			23	5.00 (2.60)			[158]
IL-8	127	mean (SD)	pg/mL			24	271.00 (473.10)			[147]
IL-8	71	mean (SD)	pg/mL			28	5.00 (1.80)	37	6.10 (2.90)	[56]
IL-8	87	mean (SD)	log pg/mL			23	0.72 (0.14)	33	0.77 (0.13)	[38]
IL-8	56	median (25^th^, 75^th^ percentile)	pg/mL	9-11	5.20 (0.90, 112.60)			29-31	4.40 (0.90, 145.90)	[36]
IL-8	28	median (25^th^, 75^th^ percentile)	pg/mL	9-11	5.30 (3.40, 7.70)			29-31	4.50 (2.20, 6.50)	[37]
IL-8	564	median (10^th^, 90^th^ percentile)	pg/mL					30	3.40 (1.80, 8.70)	[172]
IL-8	64	median (IQR)	pg/mL					31	6.00 (7.00)	[148]
IL-8	1,494	median (25^th^, 75^th^ percentile)	pg/mL					32	10.40 (3.20, 64.70)	[170]
IL-8	≤117	median (range)	pg/mL					30-33	3.50 (0.33-60.24)	[25]
IL-8	60	median (IQR)	pg/mL					36	24.50 (16.00-68.50)	[79]
IL-8	60	median (25^th^, 75^th^ percentile)	pg/mL					36	24.50 (16.00, 68.50)	[24]
IL-8	105	mean (SD)	pg/mL					36	232.10 (328.60)	[147]
IL-8	52	mean (SD)	pg/mL					UR	69.34 (111.53)	[177]

IL-12	250	median (IQR)	pg/mL	11	4.00 (11.00)					[143]
IL-12	178	median (range)	pg/mL	11	4.00 (4.00-1218.00)					[58]
IL-12	297	median (range)	pg/mL			16	4.00 (4.00-4000.00)			[77]
IL-12	392	median (IQR)	pg/mL			17	4.00 (10.00)			[143]
IL-12	233	median (range)	pg/mL			17	4.00 (4.00-276.00)			[58]
IL-12	105	mean (SD)	pg/mL			18	124.56 (95.98)			[144]
IL-12	<139	median	pg/mL			15-19	24.10			[145]
IL-12	78	median (IQR)	pg/mL			12-16	11.70 (1.80-42.75)			[146]
IL-12	56	median (25^th^, 75^th^ percentile)	pg/mL	9-11	5.60 (0.10, 70.60)			29-31	4.90 (0.00, 29.40)	[36]
IL-12	28	median (25^th^, 75^th^ percentile)	pg/mL	9-11	4.50 (2.00, 9.10)			29-31	3.00 (1.50, 7.20)	[37]
IL-12	64	median (IQR)	pg/mL					31	1.90 (4.20)	[148]

IL-12p70	71	median (range)	pg/mL	7-10	3.25 (0.00-83.44)					[142]
IL-12p70	77	median (25^th^, 75^th^ percentile)	pg/mL	10	3.10 (3.10, 14.60)					[96]
IL-12p70	504	median (25^th^, 75^th^ percentile)	pg/mL	11-13	26.50 (20.80, 34.80)					[42]
IL-12p70	104	median (25^th^, 75^th^ percentile)	pg/mL	10	3.10 (1.80, 5.50)					[43]
IL-12p70	96	median (25^th^, 75^th^ percentile)	pg/mL	12	3.30 (2.00, 5.50)					[43]
IL-12p70	93	median (25^th^, 75^th^ percentile)	pg/mL			19	2.90 (1.90, 5.90)			[43]
IL-12p70	101	median (25^th^, 75^th^ percentile)	pg/mL			24	3.30 (1.90, 6.90)			[43]
IL-12p70	100	mean (SD)	pg/mL			16	1.70 (0.80)			[154]
IL-12p70	>700	median (range)	pg/mL			15-20	1.24 (0.01-413.60)			[51]
IL-12p70	20	median (range)	pg/mL			23-25	1.60 (0.10-2.90)	32-36	1.90 (0.30-3.00)	[66]
IL-12p70	≤117	median (range)	pg/mL					30-33	1.48 (0.06-86.04)	[25]
IL-12p70	60	median (IQR)	pg/mL					36	5.00 (4.00-5.00)	[79]
IL-12p70	60	median (25^th^, 75^th^ percentile)	pg/mL					36	5.00 (4.00, 5.00)	[24]

IL-12p40	77	median (25^th^, 75^th^ percentile)	pg/mL	10	3.10 (3.10, 7.50)					[96]
IL-12p40	>700	median (range)	pg/mL			15-20	4.43 (0.01-232.23)			[78]
IL-12p40	60	median (IQR)	pg/mL					36	136.00 (118.00-168.00)	[79]
IL-12p40	60	median (25^th^, 75^th^ percentile)	pg/mL					36	136.00 (118.00, 168.00)	[24]

IL-17	13	mean	pg/mL	10-12	14.61	24-26	21.40	36-38	37.28	[72]
IL-17	≤40	median (IQR)	pg/mL	12	291.00 (66.00, 593.00)	19	112.00 (<4.00, 778.00)	33	198.00 (115.00, 524.00)	[73]
IL-17	≤40	median (IQR)	pg/mL			26	253.00 (134.00, 461.00)	39	180.00 (143.00, 259.00)	[73]
IL-17	77	median (25^th^, 75^th^ percentile)	pg/mL	10	4.30 (3.00, 10.40)					[96]
IL-17	504	Median (25^th^, 75^th^ percentile)	pg/mL	11-13	97.90 (80.20, 123.00)					[42]
IL-17	104	median (25^th^, 75^th^ percentile)	pg/mL	10	41.30 (32.20, 49.50)					[43]
IL-17	96	median (25^th^, 75^th^ percentile)	pg/mL	12	41.30 (34.00, 49.40)					[43]
IL-17	93	median (25^th^, 75^th^ percentile)	pg/mL			19	37.40 (30.50, 45.00)			[43]
IL-17	101	median (25^th^, 75^th^ percentile)	pg/mL			24	38.90 (29.70, 49.70)			[43]
IL-17	>700	median (range)	pg/mL			15-20	0.71 (0.01-46.74)			[78]
IL-17	20	median (range)	pg/mL			23-25	3.40 (0.10-12.00)	32-36	2.40 (0.10-12.70)	[66]
IL-17	≤23	median (IQR)	pg/mL			24-27	5.14 (2.73-25.69)	29-32	3.41 (2.04-23.60)	[70]
IL-17	60	median (IQR)	pg/mL					36	0.00 (0.00-0.00)	[186]
IL-17	50	mean (SD)	pg/mL					37	16.45 (4.54)	[185]
IL-17	50	mean (SEM)	pg/mL					37	773.00 (36.20)	[106]

IL-18	297	median (range)	pg/mL			16	2.50 (0.24-11.00)			[77]
IL-18	105	mean (SD)	pg/mL			18	499.86 (174.40)			[144]
IL-18	≤117	median (range)	pg/mL					30-33	71.94 (17.18-224.85)	[25]
IL-18	60	median (IQR)	pg/mL					36	56.00 (44.00-73.70)	[79]
IL-18	60	median (25^th^, 75^th^ percentile)	pg/mL					36	56.00 (44.00, 73.70)	[24]

IL-33	159	mean (SEM)	pg/mL	6	2708.00 (1805.00)					[95]
IL-33	159	mean (SEM)	pg/mL	7	4497.00 (2342.00)					[95]
IL-33	159	mean (SEM)	pg/mL	8	4862.00 (2134.00)					[95]
IL-33	159	mean (SEM)	pg/mL	9	1624.00 (731.00)					[95]
IL-33	159	mean (SEM)	pg/mL	10	1894.00 (860.00)					[95]
IL-33	159	mean (SEM)	pg/mL	11	1037.00 (574.00)					[95]

TGF-*β*	120	median (Q1-Q3)	pg/mL	0-12	473.40 (398.00-580.50)	13-27	310.40 (258.40-379.60)	≥28	325.10 (279.40-371.50)	[92]
TGF-*β*	250	median (IQR)	pg/mL	11	665.00 (1030.00)					[143]
TGF-*β*	178	median (range)	pg/mL	11	604.00 (39.00-5501.00)					[58]
TGF-*β*	297	median (range)	pg/mL			16	821.00 (39.00-13226.00)			[77]
TGF-*β*	392	median (IQR)	pg/mL			17	225.00 (1083.00)			[143]
TGF-*β*	233	median (range)	pg/mL			17	872.00 (39.00-3765.00)			[58]
TGF-*β*	105	mean (SD)	pg/mL			18	2.15 (1.50)			[144]
TGF-*β*	194	mean (SD)	pg/mL			22-25	1,288,991.00 (4,235,398.00)			[187]
TGF-*β*	50	mean (SD)	pg/mL					37	33.25 (16.74)	[185]
TGF-*β*	60	median (IQR)	pg/mL					36	364.00 (307.00-413.00)	[79]
TGF-*β*	60	median (25^th^, 75^th^ percentile)	pg/mL					36	364.00 (307.00, 413.00)	[24]
TGF-*β*	100	mean (range)	ng/mL					30-35	47.05 (31.48-142.25)	[133]

Maternal serum cytokine concentrations obtained within trimester 1 (approx. 1-12 weeks), trimester 2 (approx. 13-27 weeks), and trimester 3 (approx. 28 weeks—labour). GA: gestational age at sample collection; UR: information unreported in original manuscript; IQR: interquartile range; SD: standard deviation; SE: standard error.

**Table 2:** Anti-inflammatory cytokines measured in maternal serum in healthy pregnancy.

	N.	Concentration units		Trimester 1		Trimester 2		Trimester 3		Ref.
				GA	Concentration	GA	Concentration	GA	Concentration	
IL-4	20	mean (SD)	pg/mL	UR	0.39 (0.10)	UR	0.42 (0.17)	UR	0.39 (0.14)	[53]
IL-4	168	mean (SE)	pg/mL	7	2.00 (0.30)	20	1.30 (0.30)			[94]
IL-4	71	median (range)	pg/mL	7-10	2.81 (0.40-12.31)					[142]
IL-4	77	median (25^th^, 75^th^ percentile)	pg/mL	10	45.10 (26.80, 81.00)					[96]
IL-4	504	median (25^th^, 75^th^ percentile)	pg/mL	11-13	5.14 (4.64-5.69)					[42]
IL-4	250	median (IQR)	pg/mL	11	21.50 (70.00)					[143]
IL-4	178	median (range)	pg/mL	11	21.00 (4.00-2063.00)					[58]
IL-4	55	mean (SD)	pg/mL	12	12.30 (37.30)					[147]
IL-4	104	median (25^th^, 75^th^ percentile)	pg/mL	10	3.00 (2.60-3.50)					[43]
IL-4	96	median (25^th^, 75^th^ percentile)	pg/mL	12	3.00 (2.60-3.50)					[43]
IL-4	93	median (25^th^, 75^th^ percentile)	pg/mL			19	3.00 (2.50-3.60)			[43]
IL-4	101	median (25^th^, 75^th^ percentile)	pg/mL			24	3.00 (2.60-3.30)			[43]
IL-4	78	median (IQR)	pg/mL			12-16	2.25 (1.01-7.76)			[146]
IL-4	297	median (range)	pg/mL			16	21.00 (4.00-2156.00)			[77]
IL-4	392	median (IQR)	pg/mL			17	20.00 (71.00)			[143]
IL-4	233	median (range)	pg/mL			17	21.00 (4.00-2538.00)			[58]
IL-4	>700	median (range)	pg/mL			15-20	426.15 (26.56-2311.44)			[78]
IL-4	204	mean (SD)	log pg/mL			14-18	0.01 (1.85)			[99]
IL-4	162	mean (SE)	pg/mL			14-18	3.78 (0.38)			[97]
IL-4	<139	median	pg/mL			15-19	12.80			[145]
IL-4	61	mean (SD)	pg/mL			24	7.77 (18.90)			[147]
IL-4	1158	mean (SD)	pg/mL					28	0.15 (0.58)	[149]
IL-4	718	mean (SD)	pg/mL					28	0.14 (0.64)	[150]
IL-4	64	median (IQR)	pg/mL					31	1.80 (3.90)	[148]
IL-4	60	median (IQR)	pg/mL					36	2.00 (2.00-2.00)	[79]
IL-4	60	median (25^th^, 75^th^ percentile)	pg/mL					36	2.00 (2.00-2.00)	[24]
IL-4	70	mean (SD)	pg/mL					36	5.81 (8.67)	[147]
IL-4	50	mean (SEM)	pg/mL					37	248.00 (16.40)	[106]
IL-4	<55	mean (SD)	pg/mL					28-36	25.69 (0.11	[138]
IL-4	<139	mean (SD)	pg/mL					>37	29.25 (0.34)	[138]

IL-13	71	median (range)	pg/mL	7-10	1.85 (0.00-19.60)					[142]
IL-13	77	median (25^th^, 75^th^ percentile)	pg/mL	10	3.10 (3.10, 16.40)					[96]
IL-13	504	median (25^th^, 75^th^ percentile)	pg/mL	11-13	5.64 (4.38, 7.15)					[42]
IL-13	104	median (25^th^, 75^th^ percentile)	pg/mL	10	2.00 (0.60, 3.90)					[43]
IL-13	96	median (25^th^, 75^th^ percentile)	pg/mL	12	1.80 (0.50, 3.50)					[43]
IL-13	93	median (25^th^, 75^th^ percentile)	pg/mL			19	1.70 (0.70, 3.10)			[43]
IL-13	101	median (25^th^, 75^th^ percentile)	pg/mL			24	2.20 (1.00, 4.50)			[43]
IL-13	<139	median	pg/mL			15-19	14.30			[145]
IL-13	78	median (IQR)	pg/mL			12-16	12.06 (2.69-37.52)			[146]
IL-13	87	mean (SD)	log pg/mL			23	0.53 (0.36)	33	0.53 (0.35)	[38]
IL-13	64	median (IQR)	pg/mL					31	0.40 (2.00)	[148]

IL-10	46	median (IQR)	pg/mL	15	11.70 (6.70-25.20)	24	16.30 (10.80-32.90)	32	15.20 (7.40-40.30)	[51]
IL-10	120	median (Q1-Q3)	pg/mL	0-12	9.87 (7.26-12.87)	13-27	8.40 (5.57-9.95)	≥28	8.46 (5.45-13.68)	[92]
IL-10	20	mean (SD)	pg/mL	UR	5.06 (5.25)	UR	5.10 (5.74)	UR	8.48 (14.70)	[53]
IL-10	168	mean (SE)	pg/mL	7	125.80 (17.50)	20	179.30 (20.90)			[94]
IL-10	71	median (range)	pg/mL	7-10	0.66 (0.00-25.00)					[142]
IL-10	77	median (25^th^, 75^th^ percentile)	pg/mL	10	3.10 (3.10, 7.60)					[96]
IL-10	504	median (25^th^, 75^th^ percentile)	pg/mL	11-13	3.83 (2.88, 5.21)					[42]
IL-10	250	median (IQR)	pg/mL	11	42.00 (86.00)					[143]
IL-10	178	median (range)	pg/mL	11	40.00 (4.00-801.00)					[58]
IL-10	91	mean (SD)	pg/mL	12	26.80 (98.10)					[144]
IL-10	104	median (25^th^, 75^th^ percentile)	pg/mL	10	1.70 (0.80, 4.30)					[43]
IL-10	96	median (25^th^, 75^th^ percentile)	pg/mL	12	2.00 (1.20, 3.70)					[43]
IL-10	93	median (25^th^, 75^th^ percentile)	pg/mL			19	1.50 (0.90, 3.40)			[43]
IL-10	101	median (25^th^, 75^th^ percentile)	pg/mL			24	1.60 (0.80, 3.30)			[43]
IL-10	78	median (IQR)	pg/mL			12-16	10.84 (3.12-41.15)			[146]
IL-10	297	median (range)	pg/mL			16	252.00 (4.00-4000.00)			[77]
IL-10	100	mean (SD)	pg/mL			16	1.50 (1.10)			[154]
IL-10	392	median (IQR)	pg/mL			17	43.00 (97.00)			[143]
IL-10	233	median (range)	pg/mL			17	46.00 (4.00-891.00)			[58]
IL-10	143	mean (SD)	pg/mL			14-18	28.31 (3.16)			[98]
IL-10	204	mean (SD)	log pg/mL			14-18	2.47 (1.67)			[99]
IL-10	162	mean (SE)	pg/mL			14-18	86.02 (4.55)			[97]
IL-10	<139	median	pg/mL			15-19	7.93			[145]
IL-10	>700	median (range)	pg/mL			15-20	3.51 (0.01-578.51)			[78]
IL-10	178	mean (SD)	pg/mL			21	2.08 (1.48)			[161]
IL-10	167	mean (SD)	pg/mL			21	2.00 (1.30)			[162]
IL-10	91	mean (SD)	pg/mL			21	2.00 (0.50)			[181]
IL-10	60	mean (SD)	pg/mL			22	2.03 (0.39)			[164]
IL-10	190	mean (SD)	pg/mL			22-25	0.54 (1.24)			[187]
IL-10	124	mean (SD)	pg/mL			24	28.80 (97.90)			[147]
IL-10	65	median (IQR)	pg/mL			24-28	4.17 (1.57)			[160]
IL-10	20	median (range)	pg/mL			23-25	1.60 (0.10-2.30)	32-36	1.70 (0.10-2.50)	[66]
IL-10	87	mean (SD)	log pg/mL			23	0.15 (0.15)	33	0.13 (0.10)	[38]
IL-10	22	mean (SD)	pg/mL	8-12	0.70 (1.30)			28-34	6.80 (4.00)	[67]
IL-10	32	median (IQR)	pg/mL	8-12	8.16 (5.72-9.07)			28-32	6.45 (6.45-13.90)	[61]
IL-10	56	median (25^th^, 75^th^ percentile)	pg/mL	9-11	1.30 (0.00, 45.00)			29-31	1.10 (0.00, 19.10)	[36]
IL-10	28	median (25^th^, 75^th^ percentile)	pg/mL	9-11	1.30 (0.80, 3.10)			29-31	1.00 (0.40, 1.70)	[37]
IL-10	1158	mean (SD)	pg/mL					28	1.59 (7.85)	[149]
IL-10	718	mean (SD)	pg/mL					28	1.21 (1.88)	[150]
IL-10	64	median (IQR)	pg/mL					31	19.00 (38.30)	[148]
IL-10	1,494	median (25^th^, 75^th^ percentile)	pg/mL					32	2.00 (1.00, 3.70)	[170]
IL-10	≤117	median (range)	pg/mL					30-33	0.63 (0.01-35.90)	[25]
IL-10	774	median (IQR)	pg/mL					33	1.70 (2.50)	[171]
IL-10	60	median (IQR)	pg/mL					36	15.70 (14.00-19.00)	[79]
IL-10	60	median (25^th^, 75^th^ percentile)	pg/mL					36	15.70 (14.00, 19.00)	[24]
IL-10	94	mean (SD)	pg/mL					36	232.10 (328.60)	[147]
IL-10	<55	mean (SD)	pg/mL					28-36	13.40 (0.94)	[138]
IL-10	<139	mean (SD)	pg/mL					>37	19.83 (0.64)	[138]
IL-10	50	mean (SD)	pg/mL					37	37.60 (20.39)	[185]
IL-10	50	mean (SEM)	pg/mL					37	95.90 (68.00)	[106]
IL-10	52	mean (SD)	pg/mL					UR	3.94 (5.07)	[177]

Maternal serum cytokine concentrations obtained within trimester 1 (approx. 1-12 weeks), trimester 2 (approx. 13-27 weeks), and trimester 3 (approx. 28 weeks—labour). GA: gestational age at sample collection; UR: information unreported in original manuscript; IQR: interquartile range; SD: standard deviation; SE: standard error.

**Table 3:** Cytokines measured in maternal serum in pregnant women who developed preeclampsia.

	Comment	N.	Concentration units			Trimester 1		Trimester 2		Trimester 3	Ref.
					GA	Concentration	GA	Concentration	GA	Concentration	
IFN-*γ*		9	median (25^th^, 75^th^ percentile)	pg/mL	10	8.50 (3.70, 18.40)					[96]
IFN-*γ*		25	median (25^th^, 75^th^ percentile)	pg/mL	11-13	415.00 (334.00, 493.00)					[42]
IFN-*γ*		64	median (range)	pg/mL	11	4.00 (4.00-902.00)					[58]
IFN-*γ*		144	median (range)	pg/mL			17	4.00 (4.00-2450.00)			[58]
IFN-*γ*		409	median (range)	pg/mL			17	112.50 (4.00-3726.00)			[77]
IFN-*γ*		12	mean (SD)	log pg/mL			14-18	0.28 (2.27)			[99]
IFN-*γ*		14	mean (SE)	pg/mL			14-18	0.70 (0.20)			[97]
IFN-*γ*		300	mean (SEM)	pg/mL			25-36	177.20 (5.21)			[101]
IFN-*γ*		12	mean (SD)	pg/mL					33	35.95 (65.55)	[103]
IFN-*γ*		33	median (IQR)	pg/mL					35	210.00 (142.40-287.06)	[107]
IFN-*γ*		53	median	pg/mL					36	218.30	[105]
IFN-*γ*	both	53	mean (SEM)	pg/mL					37	221.00 (70.50)	[106]
IFN-*γ*		60	median (IQR)	pg/mL					37	5.00 (4.00-6.00)	[79]
IFN-*γ*		60	median (25^th^, 75^th^ percentile)	pg/mL					37	5.00 (4.00, 6.00)	[24]
IFN-*γ*		20	median (IQR)	pg/mL					UR	3.97 (3.97-18.06)	[104]
IFN-*γ*		11	median (range)	pg/mL					29	8.42 (0.36)	[188]

TNF-*α*		9	median (25^th^, 75^th^ percentile)	pg/mL	10	11.12 (6.10, 19.30)					[96]
TNF-*α*		25	median (25^th^, 75^th^ percentile)	pg/mL	11-13	60.90 (54.90, 69.20)					[42]
TNF-*α*		64	median (range)	pg/mL	11	4.00 (4.00-467.00)					[58]
TNF-*α*		144	median (range)	pg/mL			17	4.00 (4.00-599.00)			[58]
TNF-*α*		409	median (range)	pg/mL			17	4.00 (4.00-1770.00)			[77]
TNF-*α*		12	mean (SD)	log pg/mL			14-18	1.96 (1.39)			[99]
TNF-*α*		14	mean (SE)	pg/mL			14-18	73.57 (13.37)			[97]
TNF-*α*		31	mean (SD)	pg/mL			≥20	192.20 (92.90)			[117]
TNF-*α*		300	mean (SEM)	pg/mL			25-36	610.60 (66.51)			[101]
TNF-*α*		38	mean (SD)	pg/mL					≥28	855.80 (385.10)	[108]
TNF-*α*		60	mean (SE)	pg/mL					30	169.00 (11.00)	[186]
TNF-*α*	severe	60	median (range)	pg/mL					31	8.55 (2.10-121.00)	[115]
TNF-*α*	mild	61	median (range)	pg/mL					33	6.90 (3.03-10.50)	[115]
TNF-*α*		12	mean (SD)	pg/mL					33	12.90 (25.30)	[103]
TNF-*α*		30	mean (SD)	pg/mL					33	31.10 (13.08)	[113]
TNF-*α*	severe	20	median (range)	ng/dl					33	7.70 (3.40-20.30)	[116]
TNF-*α*	mild	22	median (range)	ng/dl					34	9.50 (4.10-35.50)	[116]
TNF-*α*	mild	32	median (25^th^, 75^th^ percentile)	pg/mL					35	84.90 (25.90, 28.90)	[136]
TNF-*α*	both	99	mean (SD)	pg/mL					35	26.49 (12.14)	[112]
TNF-*α*		53	median	pg/mL					36	185.20	[105]
TNF-*α*	both	53	mean (SEM)	pg/mL					37	203.00 (72.70)	[106]
TNF-*α*		60	median (IQR)	pg/mL					37	2.00 (2.00-3.00)	[79]
TNF-*α*		60	median (25^th^, 75^th^ percentile)	pg/mL					37	2.00 (2.00, 3.00)	[24]
TNF-*α*	mild	9	median (range)	pg/mL					37	8.85 (4.06-14.79)	[119]
TNF-*α*	severe	15	median (range)	pg/mL					37	15.95 (13.92-30.67)	[119]
TNF-*α*		<55	mean (SD)	pg/mL					28-36	20.16 (0.48)	[138]
TNF-*α*		<139	mean (SD)	pg/mL					>37	27.62 (0.64)	[138]
TNF-*α*		50	mean (SEM)	pg/mL					30-40	278.00 (31.59)	[114]
TNF-*α*		80	median (25^th^, 75^th^ percentile)	pg/mL					28-41	30.76 (28.63, 32.00)	[111]

IL-1*β*		9	median (25^th^, 75^th^ percentile)	pg/mL	10	2.20 (1.10, 3.20)					[96]
IL-1*β*		25	median (25^th^, 75^th^ percentile)	pg/mL	11-13	2.41 (2.08, 2.89)					[42]
IL-1*β*		64	median (range)	pg/mL	11	10.00 (4.00-98.00)					[58]
IL-1*β*		144	median (range)	pg/mL			17	9.00 (4.00-1634.00)			[58]
IL-1*β*		409	median (range)	pg/mL			17	214.50 (4.00-4000.00)			[77]
IL-1*β*		≤39	median (range)	pg/mL					30-33	0.38 (0.01-0.92)	[25]
IL-1*β*		12	mean (SD)	pg/mL					33	83.50 (107.20)	[103]
IL-1*β*	mild	32	median (25^th^, 75^th^ percentile)	pg/mL					35	1.90 (0.00, 317.00)	[136]
IL-1*β*		30	median (range)	pg/mL					35	2.10 (1.30-7.20)	[123]
IL-1*β*		60	median (IQR)	pg/mL					37	28.00 (23.00-34.00)	[79]
IL-1*β*		60	median (25^th^, 75^th^ percentile)	pg/mL					37	28.00 (23.00, 34.00)	[24]
IL-1*β*		20	median (IQR)	pg/mL					UR	0.55 (0.41-0.61)	[104]

IL-2		9	median (25^th^, 75^th^ percentile)	pg/mL	10	3.1 0 (0.80, 3.10)					[96]
IL-2		25	median (25^th^, 75^th^ percentile)	pg/mL	11-13	18.70 (15.60, 22.90)					[42]
IL-2		300	mean (SEM)	pg/mL			25-36	276.16 (8.70)			[101]
IL-2		12	mean (SD)	pg/mL					33	32.70 (65.00)	[103]
IL-2		53	median	pg/mL					36	273.20	[105]
	both	53	mean (SEM)	pg/mL					37	273.00 (4.76)	[106]
IL-2		60	median (IQR)	pg/mL					37	7.50 (5.50-12.00)	[79]
IL-2		60	median (25^th^, 75^th^ percentile)	pg/mL					37	7.50 (5.50, 12.00)	[24]
IL-2		50	mean (SEM)	pg/mL					30-40	497.00 (44.35)	[114]
IL-2		20	median (IQR)	pg/mL					UR	0.31 (0.18-0.79)	[104]

IL-6		9	median (25^th^, 75^th^ percentile)	pg/mL	10	3.50 (2.40, 11.30)					[96]
IL-6		25	median (25^th^, 75^th^ percentile)	pg/mL	11-13	9.40 (8.36, 12.53)					[42]
IL-6		64	median (range)	pg/mL	11	88.50 (4.00-4000.00)					[58]
IL-6		144	median (range)	pg/mL			17	73.00 (4.00-4000.00)			[58]
IL-6		409	median (range)	pg/mL			17	4.00 (4.00-4000.00)			[77]
IL-6		31	mean (SD)	pg/mL			≥20	4.80 (4.80)			[117]
IL-6		47	mean (SD)	pg/mL			16-27	2.62 (0.01-58.83)	28-40	4.57 (0.00-76.36)	[120]
IL-6	severe	32	median (IQR)	pg/mL					28-30	3.39 (1.39-4.37)	[122]
IL-6	severe	45	median (IQR)	pg/mL					30	1.10 (0.60-7.90)	[124]
IL-6	severe	60	median (range)	pg/mL					31	21.10 (2.80-248.90)	[115]
IL-6	mild	61	median (range)	pg/mL					33	13.60 (3.50-108.90)	[115]
IL-6		104	median (range)	pg/mL					33	11.81 (2.76-267.40)	[125]
IL-6	early onset	20	mean (SD)	pg/mL					33	40.80 (3.51)	[126]
IL-6		22	median (range)	pg/mL					33	24.49 (4.71-237.00)	[121]
IL-6		12	mean (SD)	pg/mL					33	14.90 (21.10)	[103]
IL-6	severe	20	median (range)	ng/dl					33	55.70 (34.20-263.40)	[116]
IL-6	mild	22	median (range)	ng/dl					34	50.20 (24.20-248.10)	[116]
IL-6		30	median (range)	pg/mL					35	7.90 (4.80-30.00)	[123]
IL-6		30	median (range)	pg/mL					35	7.80 (1.00-108.00)	[129]
IL-6	both	45	median (range)	pg/mL					36	423.67 (163.82-2749.05)	[128]
IL-6	late onset	21	mean (SD)	pg/mL					36	33.50 (1.63)	[126]
IL-6		53	median	pg/mL					36	147.00	[105]
IL-6	both	53	mean (SEM)	pg/mL					37	258.00 (26.70)	[106]
IL-6		80	median (25^th^, 75^th^ percentile)	pg/mL					28-41	7.57 (6.66, 8.92)	[117]
IL-6		60	median (IQR)	pg/mL					37	15.50 (12.00-32.00)	[79]
IL-6		60	median (25^th^, 75^th^ percentile)	pg/mL					37	15.50 (12.00, 32.00)	[24]
IL-6	mild	9	median (range)	pg/mL					37	69.79 (30.19-333.87)	[119]
IL-6	severe	15	median (range)	pg/mL					37	52.84 (112.89-669.79)	[119]
IL-6		50	mean (SD)	pg/mL					36	14.29 (10.11)	[182]
IL-6		20	mean (SD)	pg/mL					36	3.70 (20.10)	[127]
IL-6		<55	mean (SD)	pg/mL					28-36	22.68 (0.27)	[138]
IL-6		<139	mean (SD)	pg/mL					>37	26.03 (0.71)	[138]
IL-6		50	mean (SEM)	pg/mL					30-40	33.10 (8.39)	[114]
IL-6		20	median (IQR)	pg/mL					UR	2.60 (1.89-4.99)	[104]

IL-8		9	median (25^th^, 75^th^ percentile)	pg/mL	10	354.30 (190.30, 548.00)					[96]
IL-8		25	median (25^th^, 75^th^ percentile)	pg/mL	11-13	16.60 (14.30, 19.30)					[42]
IL-8		64	median (range)	pg/mL	11	13.00 (4.00-1458.00)					[58]
IL-8		144	median (range)	pg/mL			17	20.50 (4.00-4000.00)			[58]
IL-8		409	median (range)	pg/mL			17	4.00 (4.00-4000.00)			[77]
IL-8		12	mean (SD)	pg/mL					33	52.70 (69.60)	[103]
IL-8		60	median (IQR)	pg/mL					37	78.00 (35.00-273.00)	[79]
IL-8		≤39	median (range)	pg/mL					30-33	3.27 (0.61-6.08)	[25]
IL-8		60	median (25^th^, 75^th^ percentile)	pg/mL					37	78.00 (35.00, 273.00)	[24]
IL-8	mild	9	median (range)	pg/mL					37	140.40 (48.60-742.72)	[119]
IL-8	severe	15	median (range)	pg/mL					37	691.32 (140.40-1056.55)	[119]
IL-8		20	median (IQR)	pg/mL					UR	5.02 (2.61-9.14)	[104]

IL-12		64	median (range)	pg/mL	11	8.50 (4.00-1508.00)					[58]
IL-12		144	median (range)	pg/mL			17	4.00 (4.00-298.00)			[58]
IL-12		409	median (range)	pg/mL			17	4.00 (4.00-2764.00)			[77]

IL-12p70		9	median (25^th^, 75^th^ percentile)	pg/mL	10	3.10 (2.40, 50.50)					[96]
IL-12p70		25	median (25^th^, 75^th^ percentile)	pg/mL	11-13	25.80 (20.70, 31.60)					[42]
IL-12p70		≤39	median (range)	pg/mL					30-33	1.93 (0.67-247.64)	[25]
IL-12p70		60	median (IQR)	pg/mL					37	6.00 (5.00-8.00)	[79]
IL-12p70		60	median (25^th^, 75^th^ percentile)	pg/mL					37	6.00 (5.00, 8.00)	[24]
IL-12p70		20	median (IQR)	pg/mL					UR	11.93 (5.88-20.67)	[104]

IL-12p40		9	median (25^th^, 75^th^ percentile)	pg/mL	10	3.10 (3.10, 3.80)					[96]
IL-12p40		60	median (IQR)	pg/mL					37	185.00 (153.00-215.00)	[79]
IL-12p40		60	median (25^th^, 75^th^ percentile)	pg/mL					37	185.00 (153.00, 215.00)	[24]

IL-4		9	median (25^th^, 75^th^ percentile)	pg/mL	10	41.80 (24.00, 91.50)					[96]
IL-4		25	median (25^th^, 75^th^ percentile)	pg/mL	11-13	5.03 (4.49, 5.62)					[42]
IL-4		64	median (range)	pg/mL	11	25.00 (4.00-1064.00)					[58]
IL-4		144	median (range)	pg/mL			17	19.00 (4.00-1694.00)			[58]
IL-4		409	median (range)	pg/mL			17	19.00 (4.00-950.00)			[77]
IL-4		12	mean (SD)	log pg/mL			14-18	0.55 (1.69)			[99]
IL-4		14	mean (SE)	pg/mL			14-18	2.39 (0.71)			[97]
IL-4		12	mean (SD)	pg/mL					33	66.10 (106.50)	[103]
IL-4		33	median (IQR)	pg/mL					35	10.30 (3.33-18.35)	[107]
IL-4		60	median (IQR)	pg/mL					37	3.00 (3.00-4.00)	[79]
IL-4		60	median (25^th^, 75^th^ percentile)	pg/mL					37	3.00 (3.00, 4.00)	[24]
IL-4	both	53	mean (SEM)	pg/mL					37	258.00 (26.70)	[106]
IL-4		<55	mean (SD)	pg/mL					28-36	18.21 (0.05)	[138]
IL-4		<139	mean (SD)	pg/mL					>37	12.77 (0.81)	[138]
IL-4		20	median (IQR)	pg/mL					UR	0.14 (0.05-0.23)	[104]

IL-13		9	median (25^th^, 75^th^ percentile)	pg/mL	10	3.10 (3.10, 27.12)					[96]
IL-13		25	median (25^th^, 75^th^ percentile)	pg/mL	11-13	4.88 (3.73, 5.76)					[42]
IL-13	both	20	median	pg/ml					36	87.50	[132]
IL-13		20	median (IQR)	pg/mL					UR	1.50 (1.15-2.11)	[104]

IL-10		9	median (25^th^, 75^th^ percentile)	pg/mL	10	3.20 (3.10, 14.50)					[96]
IL-10		25	median (25^th^, 75^th^ percentile)	pg/mL	11-13	3.83 (2.60, 4.99)					[42]
IL-10		64	median (range)	pg/mL	11	44.50 (4.00-743.00)					[58]
IL-10		144	median (range)	pg/mL			17	41.50 (3.00-836.00)			[58]
IL-10		409	median (range)	pg/mL			17	225.50 (4.00-4000.00)			[77]
IL-10		12	mean (SD)	log pg/mL			14-18	2.17 (1.67)			[99]
IL-10		14	mean (SE)	pg/mL			14-18	39.21 (9.46)			[97]
IL-10	mild	38	mean (SD)	pg/mL			14-18	21.54 (0.89)			[98]
IL-10	severe	40	mean (SD)	pg/mL			14-18	14.84 (2.73)			[98]
IL-10		31	mean (SD)	pg/mL			≥20	4.00 (2.80)			[117]
IL-10		20	mean (SD)	pg/mL					28-36	13.34 (3.54)	[137]
IL-10		60	mean (SE)	pg/mL					30	23164.00 (996.00)	[189]
IL-10		≤39	median (range)	pg/mL					30-33	1.30 (0.21-3,498.65)	[25]
IL-10		12	mean (SD)	pg/mL					33	18.70 (29.00)	[103]
IL-10		33	median (IQR)	pg/mL					35	8.60 (2.39-16.66)	[107]
IL-10		50	mean (SD)	pg/mL					36	27.15 (13.24)	[185]
IL-10		<55	mean (SD)	pg/mL					28-36	11.26 (0.80)	[138]
IL-10		<139	mean (SD)	pg/mL					>37	7.66 (0.74)	[138]
IL-10		60	median (25^th^, 75^th^ percentile)	pg/mL					37	23.00 (18.00, 35.00)	[24]
IL-10		60	median (IQR)	pg/mL					37	23.00 (18.00-35.00)	[79]
IL-10	both	53	mean (SEM)	pg/mL					37	237.00 (251.00)	[106]
IL-10		20	median (IQR)	pg/mL					UR	2.06 (1.29-4.09)	[104]

IL-18		409	median (range)	pg/mL			17	2.90 (0.10-11.00)			[77]
IL-18		≤39	median (range)	pg/mL					30-33	72.45 (25.62-415.56)	[25]
IL-18		24	median (range)	pg/mL					35	159.90 (125.60-193.50)	[131]
IL-18		60	median (25^th^, 75^th^ percentile)	pg/mL					37	73.50 (55.00, 87.00)	[24]
IL-18		60	median (IQR)	pg/mL					37	73.50 (55.00-87.00)	[79]

IL-33	both	41	median (range)	pg/mL					36	0.21 (0.16–0.26)	135

TGF-*β*		64	median (range)	pg/mL	11	718.00 (39.00-3468.00)					[58]
TGF-*β*		144	median (range)	pg/mL			17	682.50 (39.00-4882.00)			[58]
TGF-*β*		409	median (range)	pg/mL			17	1008.00 (39.00-10256.00)			[77]
TGF-*β*		32	median (IQR)	ng/mL					32	15,092.00 (6,801.00-20,335.00)	[130]
TGF-*β*		60	median (IQR)	pg/mL					37	383.00 (331.00-418.00)	[79]
TGF-*β*		60	median (25^th^, 75^th^ percentile)	pg/mL					37	383.00 (331.00, 418.00)	[24]
TGF-*β*		50	mean (SD)	pg/mL					36	31.25 (15.32)	[185]
TGF-*β*	both	20	median (range)	ng/mL					36	25.90 (13.60-35.90)	[132]
TGF-*β*	both	140	median (range)	ng/mL					32-37	62.14 (22.19-152.13)	[133]

IL-17		9	median (25^th^, 75^th^ percentile)	pg/mL	10	5.20 (3.90, 10.40)					[96]
IL-17		25	median (25^th^, 75^th^ percentile)	pg/mL	11-13	95.40 (79.00, 120.10)					[42]
IL-17		31	mean (SD)	pg/mL			≥20	18.50 (10.80)			[117]
IL-17		32	median (IQR)	pg/mL					32	3.90 (2.55-5.06)	[130]
IL-17		20	median (range)	pg/mL					34	18.80 (11.20-25.00)	[190]
IL-17		50	mean (SD)	pg/mL					36	20.80 (9.15)	[185]
IL-17		59	median (IQR)	pg/mL					37	0.47 (0.00-0.53)	[186]
IL-17	both	53	mean (SEM)	pg/mL					37	785.00 (118.00)	[106]
IL-17		20	median (IQR)	pg/mL					UR	6.76 (4.39-11.79)	[104]
IL-17	both	40	mean (SD)	pg/mL					UR	12.00 (6.70)	[134]

Maternal serum cytokine concentrations obtained within trimester 1 (approx. 1-12 weeks), trimester 2 (approx. 13-27 weeks), and trimester 3 (approx. 28 weeks—labour). GA: gestational age at sample collection; UR: information unreported in original manuscript; IQR: interquartile range; SD: standard deviation; SE: standard error.

## Data Availability

The cytokine concentration data supporting this comprehensive review are from previously reported studies which have been cited. No new data was derived from this study.

## Abbreviations

IL: Interleukin
TNF: Tumour necrosis factor
IFN: Interferon
TGF: Transforming growth factor
Tregs: Regulatory T cells
Th: T helper cells.

## Supplementary Materials

Supplementary Materials Supplementary Table 1 outlines key concepts of the literature search conducted on Ovid/Medline databases.
