# Supplementary material for: Maternal Serum Cytokine Concentrations in Healthy Pregnancy and Preeclampsia
Source: J Pregnancy. 2021 Feb 23;2021:6649608. doi: 10.1155/2021/6649608 (PMC7925069; doi:10.1155/2021/6649608)
Supplement: Supplementary Materials — Supplementary Table 1 outlines key concepts of the literature search conducted on Ovid/Medline databases. [file 6649608.f1.docx]

|  | Key concepts of search | Key words (connected with “OR”) | Results |
| --- | --- | --- | --- |
| 1 | Pregnancy | Pregnan*; “Human pregnan*”; Maternal;  Gestation; “expectant mother” | 1117533 |
|  | **And** |  |  |
| 2 | Cytokines | “immune system”  inflammation  Cytokine*  “inflammatory marker*”  “inflammatory protein*”  Interleukin*  “Tumour necrosis factor”  “Interferon gamma”  “Transforming growth factor”  Lymphokine*  Monokine*  Chemokine* | 887286 |
|  | **And** |  |  |
| 3 | Th1/Th2 paradigm: T-lymphocytes, Th1, Th2, Th17 | “T helper cells”  “Th1 Th2 theory”  “cytokine shift”  Th1:Th2  Th1; Th2; Th17; IFN; TNF; “IL 1 Beta”; IL-2; IL-4; IL-5; IL-6; IL-7; IL-8; IL-9; IL-10; IL-12; IL-13; IL-15; IL-16; IL-17; IL-18; IL-28; IL-29; IL-21; IL-22; IL-23; IL-26; IL-25; IL-31; IL-33; TGF | 700719 |
|  | **And** |  |  |
| 4 | blood/ or plasma/ or platelet-rich plasma/ or serum/ or immune sera | Blood; plasma; serum; "platelet rich plasma;"  "immune sera;" sera | 4594215 |
| 5 | 1 & 2 & 3 & 4 |  | 5401 |
| 6 | Limit search 5 to human and English Language |  | 3402 |
| 7 | Limit search 6 to review articles only |  | 220 |
| 8 | 6 NOT 7 |  | 3182 |
| 9 | limit number 8 to yr="2009 - 2020" |  | **1834** |

**Supplementary Table 1.** Key concepts of literature search conducted on Ovid/Medline databases
